# Longitudinal Follow‐Up of Patients With Duchenne Muscular Dystrophy Using Quantitative ^23^Na and ^1^H MRI

**DOI:** 10.1002/jcsm.13812

**Published:** 2025-04-20

**Authors:** Teresa Gerhalter, Benjamin Marty, Lena V. Gast, Frank Roemer, Pierre‐Yves Baudin, Regina Trollmann, Michael Uder, Pierre G. Carlier, Armin M. Nagel

**Affiliations:** ^1^ Institute of Radiology University Hospital Erlangen FAU Erlangen Germany; ^2^ NMR Laboratory Neuromuscular Investigation Center, Institute of Myology Paris France; ^3^ Department of Pediatrics, Division Neuropediatrics FAU Erlangen Germany; ^4^ Saint‐Luc University Hospital Brussels Belgium; ^5^ Erasme University Hospital Brussels Belgium; ^6^ Liège State University Liège Belgium; ^7^ Division of Medical Physics in Radiology DKFZ Heidelberg Germany

**Keywords:** Duchenne muscular dystrophy, fat fraction, muscle imaging, quantitative MRI, skeletal muscle, sodium

## Abstract

**Background:**

Quantitative muscle MRI commonly evaluates disease activity and muscle wasting in Duchenne muscular dystrophy (DMD). Disturbances in ion homeostasis contribute to DMD pathophysiology, but their relationships with disease progression is unclear. ^23^Na MRI may provide insights into the disease course and treatment response. This longitudinal study assessed whether sodium levels are elevated in DMD patients regardless of fat fraction (FF) and whether baseline sodium levels influence FF changes over time. Additionally, we quantified the effect of slice selection on measured sodium values.

**Methods:**

Thirteen DMD boys (age 7.8 ± 2.4 years) underwent MRI of lower leg muscles at 3T at three visits, spaced 6 months apart. We assessed FF for disease progression and water T_2_, pH, apparent tissue sodium concentration (aTSC), and intracellular‐weighted ^23^Na signal (ICwS) for disease activity. Fourteen healthy boys (age 9.5 ± 1.7 years) underwent the same MRI protocol once. Linear regression and mixed‐effect modelling were used to examine sodium level increases and their impact on FF changes.

**Results:**

In DMD, muscles with FF < 10% exhibited significantly elevated aTSC (24.8 ± 4.6 mM vs. 14.5 ± 2.1 mM in controls, *p* < 0.001) and higher ICwS (23.6 ± 2.5 a.u. vs. 14.1 ± 2.1 a.u., *p* < 0.001). At Visit 1, FF values showed a significant negative association with aTSC (*β* = −17.30, *p* = 0.016) and ICwS (*β* = −21.02, *p* < 0.001).

The first mixed‐effect model, which assessed aTSC alone, showed no significant effect on FF progression but indicated a weak trend (*p* = 0.098). The second, more comprehensive model—incorporating also ICwS and water T_2_—revealed that FF changes were positively associated with aTSC (*p* = 0.0023) and negatively associated with ICwS and wT_2_ (*p* < 0.001 and *p* = 0.025, respectively), with ICwS showing a significant interaction with time (*p* = 0.0033).

Varying slice positioning and slice number demonstrated minimal impact on aTSC and ICwS, with low CV (2%–4%) in the mid‐belly region.

**Conclusions:**

The study demonstrates significant MRI‐based changes related to dystrophic alterations in DMD. We identified early alterations in sodium homeostasis, independent of FF. Our findings suggest that the relationship between sodium levels and FF progression is complex and may not be fully explained by total sodium measurements alone. Given the small sample size, further validation in larger cohorts is needed. Combined ^1^H and ^23^Na‐MRI may offer deeper insights into how metabolic and ionic changes interact with FF progression and overall disease activity.

**Abbreviations**
ATPase: Na^+^/K^+^‐adenosine triphosphatase
aTSCs: apparent total sodium concentrations
DMD: Duchenne muscular dystrophy
FF: fat fraction
ICwS: intracellular‐weighted ^23^Na signal
SRM: standardized response mean

## 1 Introduction

Duchenne muscular dystrophy (DMD) is a severe X‐linked recessive disorder caused by pathogenic variants in the dystrophin gene, resulting in complete or partial absence of the dystrophin protein. It is the most common neuromuscular disorder in children, with an incidence of one in 5000–6000 male births [1]. The absence of dystrophin renders muscle membranes vulnerable to progressive muscle damage, leading to muscle weakness and wasting. Affected individuals typically lose ambulation in their early teens and experience premature mortality in early adulthood due to cardiorespiratory complications [2].

The lack of dystrophin results in gradual deterioration of muscular tissue, characterized by disruption of ionic homeostasis, inflammation, modifications in energy metabolism, reduced regenerative capacity, and eventually replacement of muscle with fatty and fibrotic tissue [3]. While muscle function declines with age, the clinical trajectories among individuals with DMD display notable variability [4]. The interplay of underlying mechanisms contributing to this heterogeneity remains incompletely understood.

MRI provides a non‐invasive means to explore mechanisms of muscle tissue damage and their consequences. Quantitative MRI can depict muscle fat fraction (FF), which correlates with function and predicts functional decline and clinical milestones in DMD [5, 6, 7]. FF increases by approximately 3%–7% per year in DMD patients, with rates peaking at intermediate FFs [8]. Another measure of muscle damage is the increase of MRI‐derived transverse magnetization relaxation time of water (water T_2_), which reflects edema, inflammation, and necrosis, indicating higher water content, mobility, and disrupted microstructures. Increased water T_2_ has been associated with worse functional decline or higher FF increases in different myopathies [9, 10, 11].

Other MRI and NMR spectroscopy parameters, including phosphorus (^31^P) or proton (^1^H) NMR spectroscopy and sodium (^23^Na) MRI, show promise in monitoring early muscle damage in DMD [12, 13, 14, 15, 16, 17, 18]. These methods, sensitive to metabolic or ionic alterations, could serve as early markers of disease activity. Elevated phosphodiester and pH levels, measured in the absence of FF increases, reflect early metabolic alterations that precede fat replacement [16, 17]. Additionally, disturbances in ionic homeostasis—affecting sodium, potassium, calcium, iron, and other ions—arise from dystrophin deficiency and contributing to the dystrophic pathology [19, 20]. ^23^Na MRI studies have demonstrated increased sodium levels, even in early DMD stages [13, 14].

Sodium concentration is tightly regulated, with intracellular sodium levels (~15 mM) being significantly lower than extracellular levels (~145 mM). In DMD, ion homeostasis is compromised, marked by the reduced Na^+^‐K^+^ pump strength and elevated Na^+^ leakage [21]. The absence of dystrophin alters the expression, distribution, and gating properties of the voltage‐gated Na^+^ channels, leading to elevated Na^+^ concentrations under the sarcolemma and increased cell death. Specific Na^+^ channel blockers, such as tetrodotoxin, reverse Na^+^ overload and associated cell death [22]. Furthermore, dystrophin‐deficient cells exhibit reduced Na^+^/K^+^‐adenosine triphosphatase (ATPase) expression and activity, leading to a diminished resting potential [23, 24]. A recent computational study has suggested chronic sodium overload in DMD muscle triggers cytotoxic swelling and spontaneous fibre firing [21]. Although ^23^Na MRI detects chronic Na^+^ overload in patients with DMD, the relationship between sodium homeostasis and muscle fibre loss remains unclear.

Several critical questions remain unanswered. ^23^Na MRI is a valuable tool to for non‐invasive monitoring of sodium homeostasis disturbances, but longitudinal data on DMD are lacking. In addition, the influence of slice selection on sodium measurements is uncertain, as DMD exhibits proximodistal differences in muscle involvement that may affect sodium distribution [25]. Understanding the spatial and temporal dynamics of sodium homeostasis is crucial. Especially with disease‐modifying therapies on the horizon, reliable evaluation of disease progression and activity through imaging is crucial for tracking disease progression and evaluating therapeutic interventions.

This study aims to address these gaps by investigating sodium homeostasis in young DMD patients over 1 year using a combination of quantitative ^1^H and ^23^Na MRI methods. Specifically, we test three hypotheses: (I) sodium levels are elevated in DMD patients compared with controls, also in the absence of elevated FF; (II) higher baseline sodium concentrations predict a greater increase in FF over time; (III) the effect of slice selection on measured sodium values is negligible, supporting the reliability of ^23^Na MRI for longitudinal studies.

## 2 Materials and Methods

### 2.1 Subjects and Study Design

Our study received approval from the local ethical review board (Number: 250_16 Bc) and written informed consent was given by the legal guardians of each child.

We extended invitations for follow‐up scans to all 13 individuals diagnosed with DMD who had previously undergone MRI scans as part of our observational cohort study as described prior by Gerhalter et al. [13]. Results of measurements at Visit 1 have been also published previously [13]. All patients had a genetically confirmed diagnosis of DMD. Follow‐up MRI scans were performed at the Radiology department of the University Hospital Erlangen, Germany, from November 2017 to November 2019.

### 2.2 MRI Acquisition Protocol and Data Processing

All MRI exams were performed by the same protocol as described previously in Gerhalter et al. [13]. Briefly, data were acquired for the right lower leg on a 3‐T MR system (Magnetom Skyra, Siemens Healthineers, Erlangen, Germany) using a ^1^H 15‐channel knee coil (Siemens Healthineers, Erlangen, Germany). Scout images guided the positioning of imaging slices to ensure same positioning between visits. First, an axial multi‐gradient‐echo sequence was acquired (resolution 1.3 × 1.3 mm^2^, three echo times 2.75/3.95/5.15 ms, 64 slices with slice thickness 5 mm) for fat quantification. A flip angle of 3° and repetition time of 10 ms were used to minimize T_1_ weighting. Subsequently, the lower legs were also imaged with a multi‐spin echo sequence (resolution 1.4 × 1.4 mm^2^, repetition time 3000 ms, 32 echo times 9.5–304 ms, five slices with slice thickness 10 mm, slice gap 5 mm) to measure water T_2_ values. If the gastrocnemius muscle was not heavily replaced by fat, a voxel was placed in the middle of the gastrocnemius medialis muscle to acquire two ^1^H spectra (16 averages without water suppression and 64 averages with water suppression using PRESS localization, voxel size: 9 × 30 × 30 mm^3^, repetition time 3000 ms, echo time 30 ms, bandwidth 2000 Hz) to measure pH (Figure S1).

^23^Na images were acquired using a 1‐channel transmit/receive ^23^Na birdcage knee coil (Stark Contrast, Germany) after pulse calibration with a 3D‐radial sequence with a density‐adapted readout scheme (echo time 0.3 ms, flip angle 80°, readout duration 10 ms, resolution 3 × 3 × 15 mm^3^, interpolated with zero filling to 1 × 1 × 7 mm^3^). A repetition time of 50 ms was used to minimize the acquisition duration. Another 3D‐radial sequence was acquired with an inversion‐recovery preparation (echo time 0.3 ms, inversion time 34 ms, repetition time 124 ms, flip angle 90°, readout duration 20 ms, resolution 4 × 4 × 20 mm^3^, interpolated with zero filling to 1 × 1 × 7 mm^3^) to suppress the sodium signal from a liquid environment. ^1^H images for anatomical imaging were acquired with the body coil (echo time 4.77 ms, repetition time 308 ms, nominal resolution 1 × 1 mm^2^, slice thickness 5 mm, slice gap 2 mm).

### 2.3 Postprocessing of ^1^H MRI Data

FF and water T_2_ values were calculated using an in‐house developed Python tool using the 3‐point Dixon method on the multi‐gradient‐echo images and using a triexponential adjustment of the multi‐echo spin‐echo images model [13], respectively. Based on the shift between the carnosine and residual water resonance in the ^1^H spectra, the pH values were calculated as described in [17], if the following quality criteria were met: (i) the spectra showed a water peak linewidth below 30 Hz, and (ii) the signal to noise ratio was above 10.

The region‐of‐interests were semi‐automatically drawn on the magnitude images of the first echo of the Dixon data set using DAFNE [26]. Subsequently, they were co‐registered onto the quantitative maps. For muscle‐specific analysis, three commonly affected muscles (soleus, gastrocnemius medialis, tibialis anterior) and one relatively spared muscle (tibialis posterior) from the three middle slices of the water T_2_ map (and the corresponding slices of the other maps) were selected for statistical analysis of longitudinal changes. Averaging over several slices (3.5 cm length) should mitigate possible misplacement errors. In addition, particular attention was given to selecting the same area of the leg as DMD exhibits proximodistal differences over the length of the entire muscle [25]. Each longitudinal dataset was evaluated individually to ensure a consistent comparison of the same muscle area over time.

### 2.4 Postprocessing of ^23^Na MRI Data

Postprocessing of sodium data was performed with MATLAB (Version R2020b) using in‐house developed tools. Apparent tissue sodium concentrations (aTSCs) and intracellular‐weighted ^23^Na signal (ICwS) maps were derived from the ^23^Na images acquired without and with inversion‐recovery preparation, respectively [13]. Magnitude images were reconstructed offline using a Hamming filter to reduce ringing artefacts.

Average signal intensities were calculated from two calibration phantoms (containing 20 and 40 mmol/L NaCl in 5% agarose gel, designed to mimic the relaxation properties of muscle tissue) and from the background signal (0 mmol/L). These calculations were based on the five middle slices of the ^23^Na images. A linear regression curve was generated from the phantom data and used to extrapolate aTSC and ICwS maps, respectively. Values were subsequently corrected for relaxation effects [27] and fat replacement based on the Dixon‐derived FF (Figures S2 and S3) [13]. Given that muscles with low FF usually do not exhibit strong proximodistal pattern, particularly in the mid‐belly region [25], sodium values were adjusted using a global FF derived from the 3.5 cm muscle section. To evaluate longitudinal changes, we averaged the mean sodium values (aTSC and ICwS) across the five middle slices.

### 2.5 Slice‐Based Variability Analysis for ^23^Na MRI Data

Variations in sodium values across the five central slices of the ^23^Na datasets were analysed to assess slice‐dependent changes. First, changes in the slice position were assessed with two locations along the proximodistal muscle axis. At the first location, mean aTSC and ICwS were averaged over four central slices from the thickest part of the calf. At the second location, one slice proximal to the first location were analysed, covering an equivalent distance (2.8 cm) along the proximodistal axis. Additionally, single‐slice average values were computed for the central slice of each muscle region to facilitate comparison with the five‐slice analysis.

### 2.6 Statistical Analysis

Statistical analysis was performed with MATLAB (Version R2020b). Demographics were expressed as means ± standard deviation and range, and MRI results as median [first quartile − third quartile], as most data were non‐normally distributed. Statistical significance was set at *p* < 0.05, unless otherwise stated.

To test Hypothesis 1, the difference in sodium levels between DMD muscle at baseline with FF < 10% and control (CTL) muscles was evaluated using the Wilcoxon rank‐sum test. A Bonferroni correction was applied to correct for multiple comparisons resulting in a significance level of *p <* 0.0125. In addition, linear regression models were employed, with sodium levels (aTSC or ICwS) at Visit 1 as the dependent variable. Disease status (DMD vs. CTL) and FF were included as independent variables, while individual muscles were modelled as categorical covariate to account for repeated measures.

For Hypothesis 2, changes in FF over time were quantified using the standardized response mean (SRM), calculated as the ratio of the observed change to the standard deviation of the change. An SRM of 0.8 or greater was interpreted as high responsiveness. To assess FF changes over time for all three visits, the linear model was used, *FatFraction ~ Days + Muscle*, where ‘Muscle’ refers to the different muscle groups (soleus, gastrocnemius medialis, tibialis anterior and posterior), and ‘Days’ refers to the number of days since the first visit. Next, scatterplots were used to visualize the relationship between FF changes and baseline sodium and water T_2_ levels. Spearman's rank correlation coefficients (rho) were calculated for exploratory analyses to assess the strength of these associations. To explore whether baseline sodium levels influenced FF progression, we applied two mixed‐effects models.

1. Sodium and FF progression: The first model, *FatFraction ~ 1 + Muscle + Days* * *aTSC*, examined the effect of total sodium (aTSC) on FF changes over time. Here, ‘Muscle’ refers to the different muscle groups, ‘Days’ corresponds to the number of days since the first visit, and ‘aTSC’ represents sodium levels. The interaction term (*Days* * *aTSC*) was included to evaluate whether the progression of FF over time was modulated by baseline sodium levels.
2. Extended model with sodium homeostasis and water T_2_: Given the complexity of sodium regulation, we developed a second, more comprehensive model, *FatFraction ~ aTSC* * *Days + ICwS* * *Days + wT*_*2*_ * *Days + Muscle*, to account for additional factors that may influence FF changes. This model incorporated intracellular sodium‐weighted signal (ICwS) and water T_2_ (wT_2_), which reflect edema‐related changes and thus changes in the volume fraction. These variables were included as interaction terms with ‘Days’ to assess their potential contributions to FF progression.

Due to the limited number of study participants, individual slopes per subject were not included in the models.

Hypothesis 3 was tested by assessing the influence of slice selection on sodium measurements. Bland–Altman plots were used to assess the difference between aTSC and ICwS calculated at the central slice versus the average of five central slices. Coefficients of variation (CV) were calculated to evaluate variability when slices were shifted and between single‐slice and 5‐slice averages.

## 3 Results

### 3.1 Characteristics and Demographics of Longitudinal Study Participants

**FIGURE 1:**
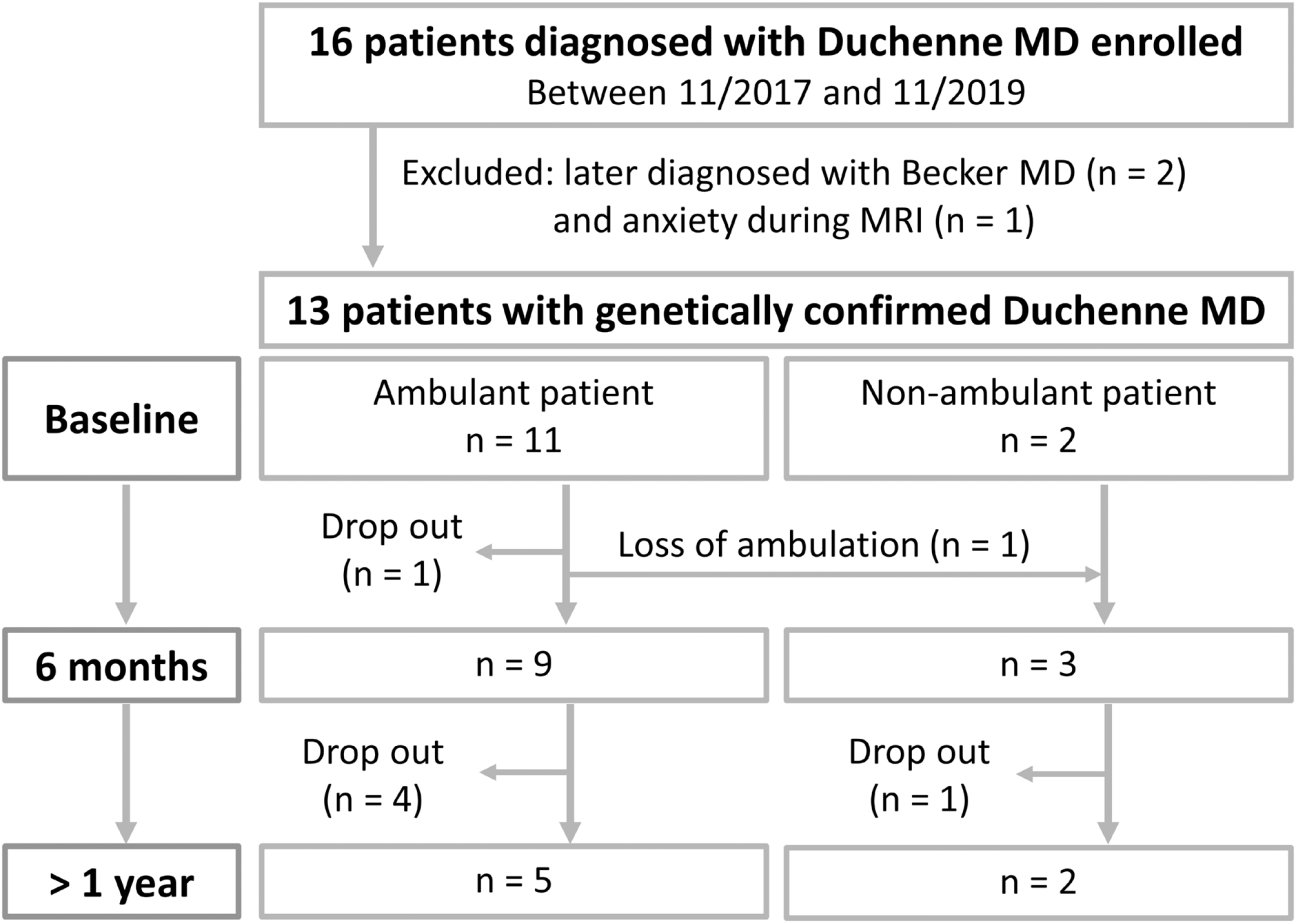
Flow diagram depicting study design, enrollment and dropouts. Patients were recruited through the local Children's Hospital and through newsletters distributed by the patient registry of the Ludwig‐Maximilians‐Universität Munich. Eligible patients who were willing underwent up to three MRI scans with a 6‐month interval between scans. MD, muscle dystrophy.

Of the 13 patients from the previous case–control study [13], all but one were scanned 28.1 ± 5.5 weeks after the first visit (Figure 1). Five patients participated in their second follow‐up at 54.4 ± 3.8 weeks, while two had their second follow‐up at 84 weeks due to scheduling difficulties. Additionally, 14 age‐matched male controls were scanned once. Table 1 summarizes the participant's demographic and characteristic. The average age of participants with DMD was 7.8 ± 2.4 years at their first visit, with all but two still ambulant (defined as walking > 20 m without assistance). One patient lost ambulation before the first follow‐up. Table S1 contains medication details for each visit and patient's mutation.

**TABLE 1:** Summary of subject demographics and characteristics. Ambulation was defined as walking for 20 m without assistance. Medication was recorded if the patient was on treatment at the time of the MRI scan. Medications at Visit 1 consisted of steroids (*n* = 6), ataluren (*n* = 2), L‐citrulline and metformin (*n* = 2), and idebenone (*n* = 1). Wilcoxon rank sum tests (WRST) tested equality between control and DMD populations and Wilcoxon signed rank test (WSRT) differences between the first visit and follow‐up visits for patients.

	CTL (*n* = 14)	DMD (*n* = 13) Visit 1^a^	WRST	DMD (*n* = 12) Visit 2	WSRT	DMD (*n* = 7) Visit 3	WSRT
Age, years Mean ± std [range]	9.1 ± 1.7 [6.5–11.4]	7.8 ± 2.4 [5–11.5]	*p* = 0.14	8.6 ± 3.3 [5.5–12]		9.8 ± 5.5 [6.6–12.5]	
Weight, kg Mean ± std [range]	32.2 ± 7.4 [21–47]	28.5 ± 12.9 [18–51]	*p* = 0.13	31 ± 14.7 [21–59]	*p* = 0.13	37.1 ± 22.5 [24–67]	*p* = 0.02
Height, cm Mean ± std [range]	137.6 ± 12.2 [122–158]	124.2 ± 13.2 [105–148]	*p* = 0.01	126.8 ± 37.3 [110–149]	*p* = 0.06	131.6 ± 68.9 [115–150]	*p* = 0.06
BMI Mean ± std	17 ± 2.5	17.8 ± 4.6	*p* = 0.7	18.7 ± 6.6	*p* = 0.6	20.8 ± 11.7	*p* = 0.08
Ambulant Yes/no	14/0	11/2		9/3		5/2	
Medication Yes/no	0/14	8/5		9/3		6/1	

^a^ Data from Visit 1 was previously published in [13].

### 3.2 Elevated Sodium and FF in DMD Muscles at Baseline

**FIGURE 2:**
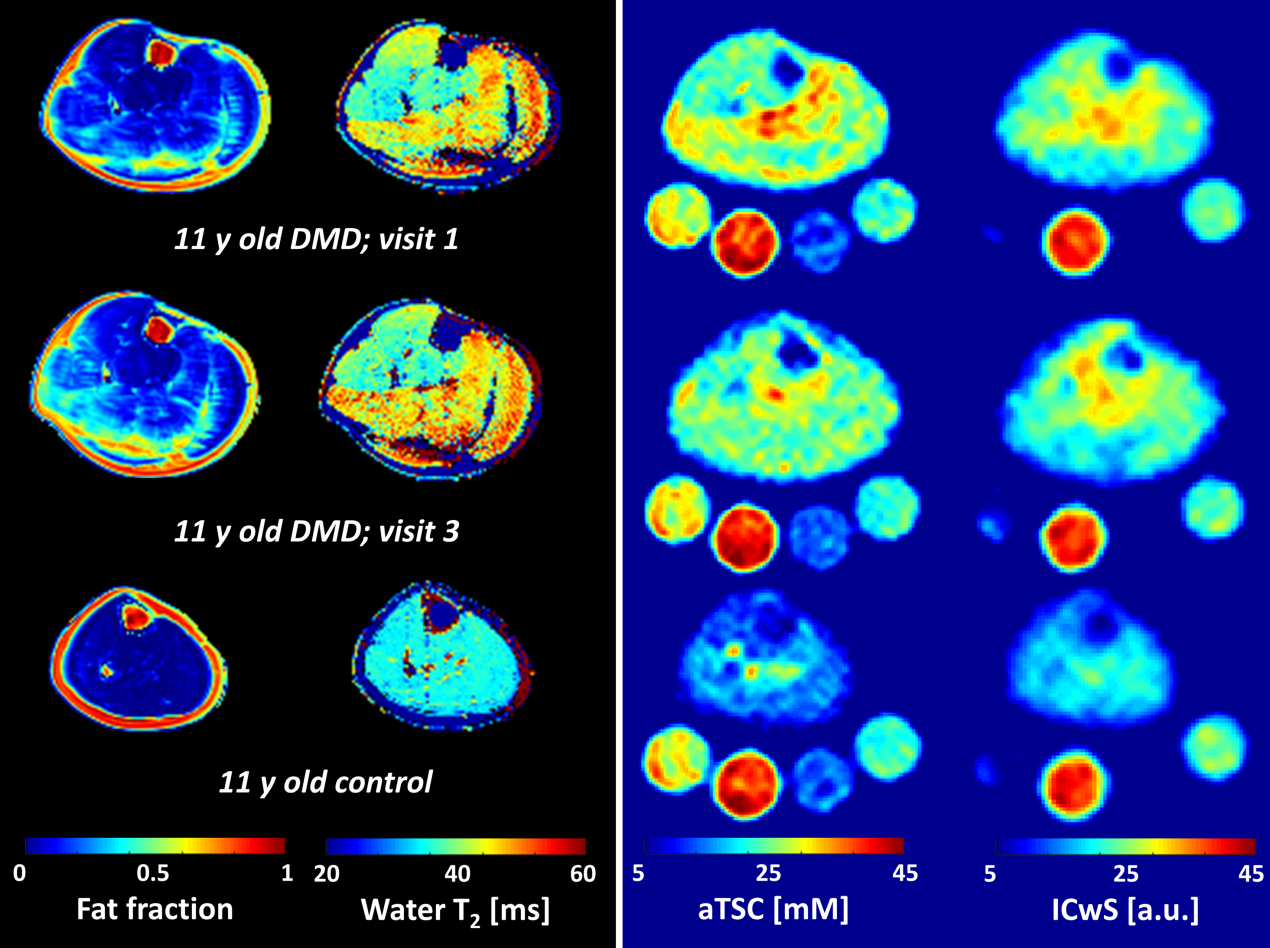
Quantitative MRI‐derived muscle quality maps of the lower leg of an 11‐year‐old patient at Visit 1 (first row) and Visit 3 (second row) and an age‐matched control (third row). Dystrophic muscles showed increased fat content, water T_2_, apparent tissue sodium concentration (aTSC), and intracellular‐weighted sodium signal (ICwS) compared with healthy muscle tissue. Active disease activity, as indicated by higher water T_2_, aTSC, and ICwS, was also observed in spared muscles with low fat content, such as the tibialis anterior and tibialis posterior muscles. The legs are positioned on top of reference phantoms that are utilized for ^23^Na signal quantification (from left to right: 40 mM NaCl, 40 mM NaCl + 5% Agarose, 20 mM NaCl, 20 mM NaCl + 5% Agarose). It is worth noting that the inversion‐recovery pulse effectively suppresses the ^23^Na signal from the saline solution (without agarose) in the ICwS images.

Figure 2 displays quantitative MRI maps acquired from the lower legs of an 11‐year‐old patient at his first visit, 1‐year follow‐up, and a healthy control. Compared with healthy muscle tissue, dystrophic muscles exhibited increased FF, water T_2_, aTSC, and ICwS. Changes in FF over time were evident in the comparison of FF maps collected at Visits 1 and 3 for this patient, with notable increases observed in nearly all muscles, particularly the soleus, peroneus, and extensor digitorum longus. Moreover, markers of active disease activity—including higher water T_2_, aTSC, and ICwS—were present in this patient, even in spared muscles with low FF (e.g., tibialis anterior and tibialis posterior muscles).

Table S2 summarizes the MRI‐derived values for the four investigated muscles. At Visit 1, dystrophic muscles demonstrated significantly higher FF, wT2, aTSC, ICwS, and pH compared with control muscles, as reported previously [13]. Specifically, in muscles with FF < 10%, aTSC was significantly elevated in DMD compared with control muscles (24.8 ± 4.6 mM vs. 14.5 ± 2.1 mM, *p* < 0.001). Similarly, ICwS was higher in DMD muscles (23.6 ± 2.5 a.u. vs. 14.1 ± 2.1 a.u., *p* < 0.001). To further analyse these differences, linear regression models were employed to investigate the effects of disease status, muscle category, and FF on sodium concentration (Table S3). For aTSC, disease status was a significant predictor (*β* = 10.74, *p* < 0.001), while FF showed a significant negative association (*β* = −17.30, *p* = 0.016). The model explained 73% of the variability in aTSC (*R* ^2^ = 0.73, *p* < 0.001). For ICwS, a similar pattern was observed. Disease status was a highly significant predictor (*β* = 9.76, *p* < 0.001), and FF was negatively associated with ICwS (*β* = −21.02, *p* < 0.001). The ICwS model accounted for 79% of the variability (*R* ^2^ = 0.79, *p* < 0.001).

### 3.3 Associations Between Baseline Sodium Levels and FF Progression

**FIGURE 3:**
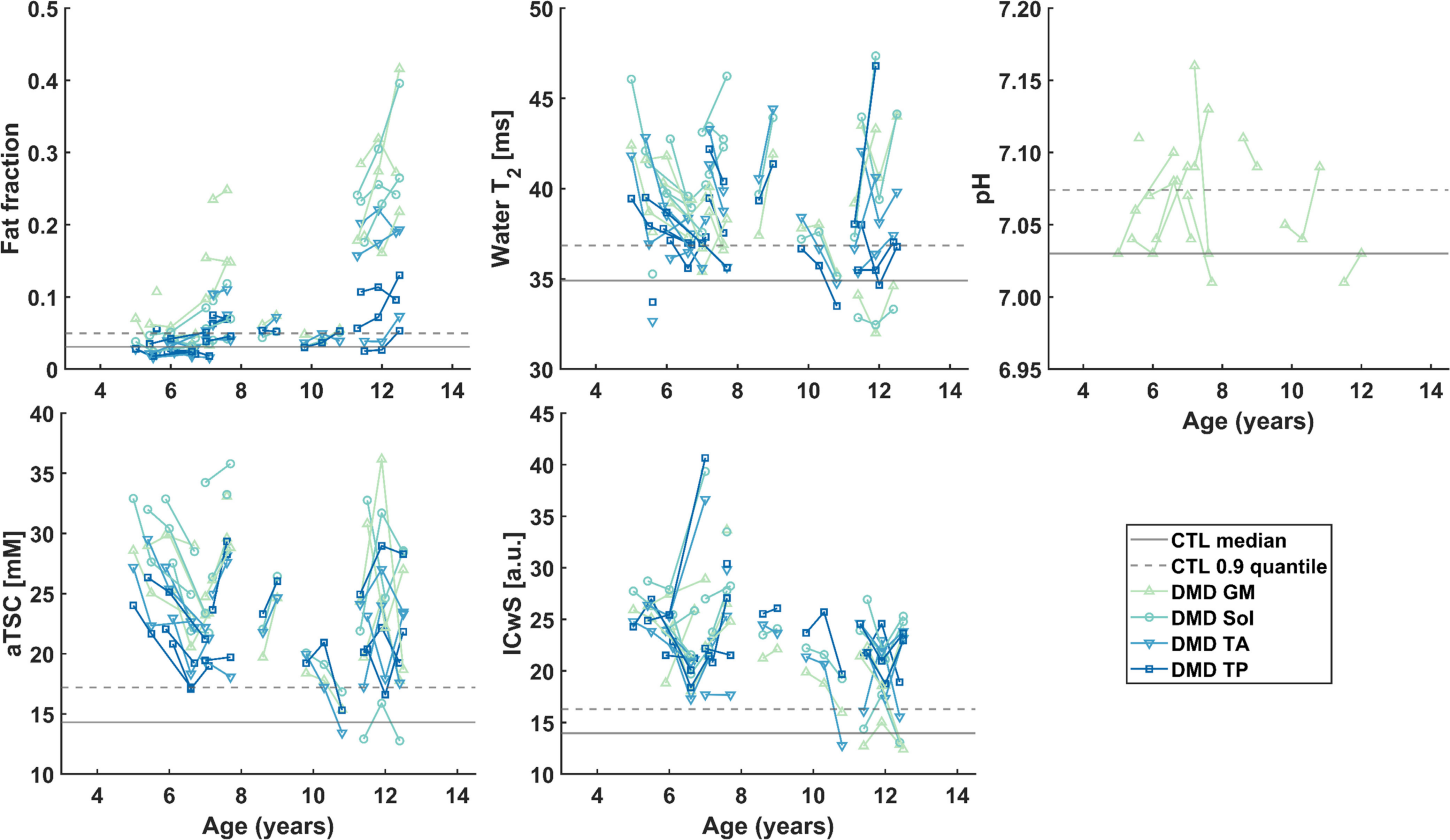
Changes over time for quantitative MRI derived measures. Each line represents a single muscle over age, with different colours indicating the four different muscles. Dystrophic muscles showed an overall increase in fat fraction, water T_2_, aTSC, and ICwS compared with healthy muscle tissue (median across all four muscles shown with a solid grey line and the corresponding 0.9 quantile with a dashed grey line), but no difference in pH. CTL, control; GM, gastrocnemius medialis; Sol, soleus; TA, tibialis anterior; TP, tibialis posterior.

Figure 3 illustrates the longitudinal trajectories of MRI‐derived measurements for the investigated muscles. While FF generally increases with age and over time, disease progression varied considerably between individuals and muscles. Between Visits 1 and 2, global FF increased by an average of 0.006 [0.0003–0.013] (*p* = 0.077, SRM = 0.49), with the most pronounced increase observed in the soleus muscle (0.008 [0.001–0.023], *p* = 0.036, SRM = 0.65). Among the seven patients who returned for a third visit, global FF increased further on average by 0.013 [−0.006 to 0.042], *p* = 0.297, SRM = 0.55) (Table S3). In contrast, mixed‐effects modelling of FF changes over time revealed a significant intercept (*p* < 0.001) but no significant effect of time on FF changes (*p* = 0.25). Muscle group had a significant effect, with the soleus and gastrocnemius muscles showing greater FF increases compared with the tibialis anterior (Table S4). During the same period, markers of active disease, including water T_2_, aTSC, and ICwS, remained elevated in patients compared with healthy controls (Table S3).

**FIGURE 4:**
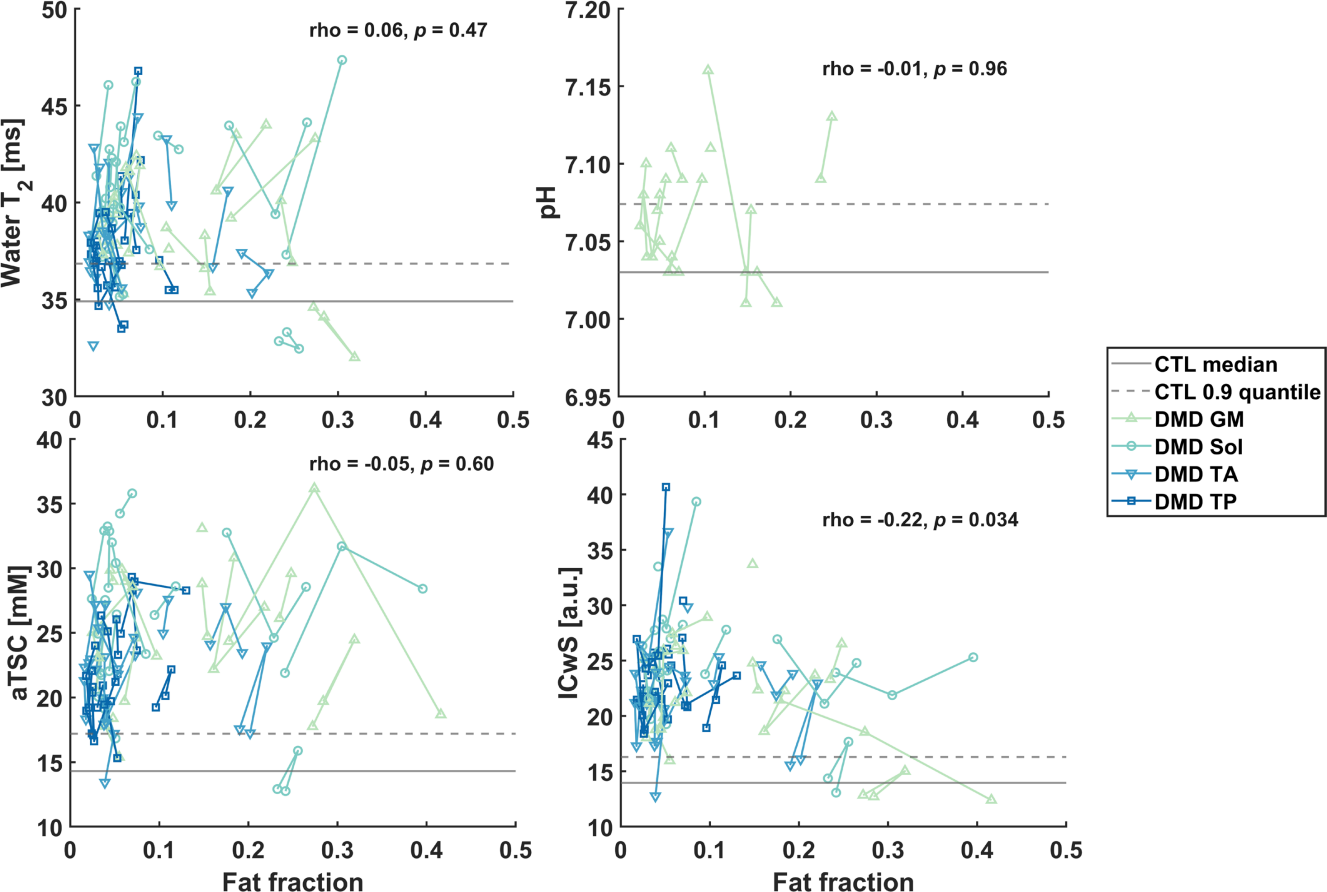
Water T_2_, pH, aTSC, and ICwS plotted against fat fraction for the three visits. Each line represents a single muscle over time, with different colours indicating the four different muscles. Dystrophic muscles showed increased water T_2_, aTSC, and ICwS compared with controls, with their median across all four muscles shown as a solid grey line and the 0.9 quantile as a dashed line. These increases in water T_2_, aTSC, and ICwS were also observed in muscles with low fat fraction. ICwS demonstrated a negative correlation with fat fraction (Spearman rho = −0.22, *p* = 0.034). Water T_2_ values are missing for one patient (high fat fraction) for Visit 3, as the leg was too stiff to correctly position the calf in the isocenter of the coil. CTL, control; GM, gastrocnemius medialis; Sol, soleus; TA, tibialis anterior; TP, tibialis posterior.

Across all visits, dystrophic muscles exhibited high water T_2_, aTSC, and ICwS in muscles with low to medium FF (Figure 4). Spearman's rank correlation analysis revealed a significant negative correlation between baseline ICwS and FF (rho = −0.22, *p* = 0.034), suggesting that higher intracellular sodium levels were associated with lower FF.

Scatter plots of FF changes over time versus disease activity did not show clear correlations between FF increases and baseline water T_2_, aTSC, or ICwS (Figure S6). To explore the longitudinal effects solely of baseline sodium levels on FF, we applied a mixed‐effects model (Table S4). Results indicated no direct effect of baseline sodium (aTSC) on FF progression (*p* = 0.18). However, the interaction between time (Days) and aTSC approached significance (*p* = 0.098), suggesting that higher baseline sodium levels might slightly influence FF changes over time.

To account for the complexity of sodium homeostasis, we applied a second, more comprehensive model that included additionally ICwS and water T_2_ (Table S4). This model identified significant predictors of FF changes over time. Specifically, aTSC was positively associated with FF (*p* = 0.0023), while ICwS and wT_2_ were negatively associated with FF (*p* < 0.001 and *p* = 0.0251, respectively). Additionally, the interaction between ICwS and Days was significant (*p* = 0.0033), indicating that higher ICwS at baseline influences FF changes over time.

### 3.4 Differences in Sodium Values due to Positioning Shifts and Slice Selection

Shifting the analysed slices by one slice in the proximal direction led to a significant difference in ICwS values but not in aTSC (Table 2). Specifically, the average change in aTSC was −0.17 mM in patients (maximum: −1.7 mM) and −0.06 mM in controls (maximum: −1.6 mM). In contrast, ICwS exhibited a more pronounced average change of −0.48 a.u. in patients (maximum: −1.35 a.u.) and −0.25 a.u. in controls (maximum: −1.2 a.u.). CVs across the five middle slices range between 2% and 6% (Figure S4). When quantifying sodium values based solely on the central slice instead of averaging across five slices, Bland–Altman analysis revealed negligible changes in aTSC and ICwS. aTSC showed a reproducibility coefficient of 0.72 mM (4.0%), and ICwS showed a reproducibility coefficient of 0.64 a.u. (4.1%) (Figure S5).

**TABLE 2:** Median with the first and third quartiles of the difference in apparent total tissue sodium concentration (aTSC) and intracellular‐weighted sodium signal (ICwS) for a one slice shift in position along the proximodistal axis. Differences are presented for patients with DMD and for controls. Significant differences are marked with †.

	Muscle	Control			DMD Visit 1		
		Median	Q1	Q3	Median	Q1	Q3
aTSC	Soleus	0.03	−0.18	0.18	0.05	−0.20	0.41
	Gastrocnemius medialis	0.09	−0.25	0.22	−0.10	−0.53	0.16
	Tibialis anterior	−0.07	−0.67	0.11	−0.31	−0.54	0.21
	Tibialis posterior	−0.17	−0.61	−0.04	−0.26	−0.89	−0.16
	Mean	−0.06	−0.42	0.17	−0.17	−0.46	0.25
ICwS	Soleus	−0.30 †	−0.59	−0.13	−0.33 †	−0.47	−0.20
	Gastrocnemius medialis	−0.24 †	−0.50	−0.10	−0.72 †	−0.93	−0.47
	Tibialis anterior	−0.24 †	−0.55	−0.02	−0.62 †	−0.86	−0.28
	Tibialis posterior	−0.22 †	−0.69	−0.08	−0.43 †	−0.98	−0.22
	Mean	−0.25 †	−0.60	−0.09	−0.48 †	−0.87	−0.23

## 4 Discussion

In this longitudinal study, we aimed to explore the utility of ^23^Na MRI for assessing the disease evolution in the lower limb muscles of patients with DMD over 1 year. We addressed three hypotheses: (I) that sodium levels are elevated in DMD patients irrespective of the FF, (II) that baseline sodium levels are associated with FF changes over time, and (III) that the effect of slice selection on measured sodium values is minimal, supporting the reliability of ^23^Na MRI for longitudinal studies. Initial observations confirmed increases in FF, water T_2_, pH, aTSC, and ICwS of patients with DMD compared with age‐ and gender‐matched healthy controls. While progression of the disease varied substantially among patients and across different muscle groups, muscles with low FF showed substantial aTSC and ICwS increases. While the first mixed‐effect model did not indicate a direct effect of baseline aTSC on FF progression, the second more extensive mixed‐effects model revealed that FF changes were influenced by both baseline sodium levels (aTSC) and intracellular sodium (ICwS), with ICwS showing a significant interaction with time. Lastly, analysis of slice positioning demonstrated low CV in the mid‐belly region of the muscle. Nevertheless, a careful selection of slices is needed for standardized acquisition protocols.

MRI has emerged as a valuable tool in clinical trials, providing sensitive and non‐invasive biomarkers for monitoring disease progression in DMD [28, 29]. Progression is mainly associated with fat replacement of the muscle tissue, which has been identified as a predictive factor for ambulation loss in DMD patients [30, 31]. Moreover, muscle architecture, particularly muscle fibre length, has been found to correlate with FF in DMD, as impaired force propagation possibly leads to contraction‐induced damage [32]. The changes in FF in our cohort varied among the analysed muscles. Our average increase was lower than the reported 3%–7% per year [8], likely due to the young age of our DMD cohort. Our findings suggest considerable variability between individuals and muscle groups. This is reflected in the descriptive analysis, where global FF increased on average by 0.013 between Visits 1 and 3, with the most pronounced increase observed in the soleus and gastrocnemius muscles. However, despite these observed increases in individual patients, our mixed‐effects model did not detect a significant overall effect of time on FF changes across the cohort. This apparent discrepancy likely results from the substantial variability in disease progression among individuals and muscles, reinforcing the importance of considering inter‐individual differences when assessing longitudinal FF changes.

Other MR indices can capture different aspects of muscle dystrophy in addition to fat replacement. Water T_2_ values for example serve as indicator of active inflammation, edema, or cell lesions in muscle tissue [33]. It is important to note that the progression of T_2_ values does not parallel that of fat replacement. Instead, water T_2_ values tend to be highest in the earlier stages of the disease [34]. Moreover, in patients undergoing corticosteroid therapy, a decrease in water T_2_ values has been observed, indicating a reduction in inflammation and suggesting the therapeutic efficacy of such interventions [35]. This study found water T_2_ is higher in patients compared with healthy controls, notably also in in low‐fat muscles. However, we did not see a correlation between water T_2_ and FF or FF changes, reflecting the complex water T_2_ dynamics.

Severe disruptions in ion homeostasis, particularly sodium dysregulation, which was first documented in patients in 1955 by Horvath and colleagues, is thought to contribute to the muscle damage and loss of cell integrity in DMD. Although histopathological data were not available in this study, prior histopathological studies confirmed increased sodium levels in dystrophic muscles in mice models and patients (Supporting Information reference list). Dysfunctional ion channels and impaired sodium pump activity lead to cytotoxic swelling and cellular dysfunction, but is not inherently lethal to the muscle fibres as they can survive for decades [21]. As previously reported [13, 36], ^23^Na MRI has measured chronic sodium overload, which can be however also non‐osmotic, i.e., not accompanied by water uptake. Computational models show that even with DMD‐related insults like low pump strength, overstimulation, and leaky sodium and cation channels, the fibres manage to survive due to a unique process called the pump‐leak/Donnan mechanism [21]. Only severe stress in skeletal muscle fibres with DMD would then lead to spontaneous firing, cytotoxic swelling, increased sodium and calcium influx, energy depletion, and exacerbation of DMD symptoms, potentially worsening muscle damage and dysfunction. Maintaining ion homeostasis is therefore thought to be crucial for preserving muscle integrity and function in DMD patients. Longitudinal monitoring of these patients with ^23^Na MRI would provide potential insights into the extent of sodium overload and ion homeostasis disruption, potentially aiding in the monitoring of disease‐modifying treatments.

Here, we tested for the first time the impact of slice selection on sodium quantification. Our study has shown that a shift of one slice (7 mm) along the proximodistal muscle axis results in a small but significant difference in ICwS, but not in aTSC. These findings highlight the potential sensitivity of ICwS to slice positioning and the importance of multi‐slice averaging for robust quantification. In comparison to previously investigated proximodistal dependency of FF [25], we only covered a quarter of the distance of the calf (13 cm vs. 3.5 cm), which might cause the relatively small changes in sodium levels over the investigated muscle length. Importantly, these changes (about 2%–4%) are much smaller than the differences between controls and patients. The small reproducibility coefficients of about 4% between 1‐slice analysis and 5‐slice analysis further highlight the sensitivity of ^23^Na MRI to track changes in longitudinal studies, provided they are big enough. In a DMD case study, aTSC dropped by 13%–30% and ICwS by 3%–31% depending on the muscle, 6 months after treatment [12]. Such interventional changes are therefore large enough to be detected by ^23^Na MRI given the current quantification accuracy.

In our cohort, aTSC and ICwS remained persistently elevated between visits and compared with controls. However, we did not observe a linear correlation between ionic disturbances and disease progression as measured by FF changes over time. This suggests that while FF accumulates with age or disease duration, metabolic and ionic MRI measures such as aTSC and ICwS may fluctuate dynamically at particular disease stages, influenced also by specific muscle groups. To explore the longitudinal effects of baseline sodium levels, we applied two different mixed‐effects models (limitations are discussed in Section 5). The first model, which assessed aTSC alone, showed no significant direct effect on FF progression but suggested a potential trend. Given the complexity of sodium homeostasis, we also applied a second model that included ICwS and water T_2_. This model identified that higher baseline ICwS, but not aTSC, significantly influenced FF changes over time. The differing results could stem from the fact that the first model only considered total sodium, while the second model accounted for intracellular sodium and edema‐related changes, both of which impact sodium homeostasis and FF progression. These findings suggest that the relationship between sodium and FF is complex and may not be fully captured by total sodium alone.

It is important to note that aTSC is a volume fraction‐weighted average of intra‐ and extracellular sodium signals. Changes in aTSC can be attributed to alterations in the concentrations of sodium within the intra‐ and extracellular compartments, as well as changes in the volume fractions of these compartments or a combination of these factors (‘Hilal ambiguity’) [37]. Our previous findings indicated strong correlations between water T_2_ and aTSC in DMD [13], which could be attributed to the interaction of water and ionic balance. In this study, we confirmed a strong correlation between water T_2_ and aTSC and, to a lesser extent, with ICwS (Figure S7). However, aTSC and ICwS do not fully reflect water T_2_. For instance, muscles with aTSC and ICwS increases do not necessarily exhibit elevated water T_2_, suggesting that these measures capture overlapping but distinct aspects of disease activity. This also supports the pump‐leak/Donnan mechanism, where muscle fibres may tolerate chronic sodium overload and only exhibit swelling under severe stress. As both water T_2_ and ^23^Na MRI signal are a mixture of different intra‐ and extracellular alterations, further studies are needed to disentangle their contributions to disease activity. First, animal studies with sodium shift reagents could clarify the specific pathological alterations in intra‐ and extracellular compartments. Second, human studies with larger cohorts, longer follow‐ups, and standardized muscle function testing are necessary to determine how these metabolic and ionic changes interact with FF progression and overall disease activity.

## 5 Study Limitations and Technical Remarks

There are some limits to this study. The patients had different DMD mutation locations leading to dystrophin deficiency. These variations may partly explain the heterogenous disease progression [38]. All patients had mutations affecting the full‐length dystrophin protein (Dp427), but two also had disrupted production of the shorter dystrophin isoform Dp140, which is highly expressed in the brain and may worsen motor function [38]. Furthermore, corticosteroid use, associated with delayed decline in muscle, ambulatory, pulmonary, and cardiac function [2], varied among participants in this natural history study based on medical counselling. We did not analyse the effects of corticosteroids or other treatments on MR measures. However, future studies should include more participants and incorporate the treatment regimen on MR measures.

Water T_2_ values were determined using the tri‐exponential fitting procedure. Although promising, recent water T_2_ mapping procedures based on the extended phase graph (EPG) algorithm were not used in this study. Instead, the tri‐exponential fitting procedure was chosen due to its validation and consistency with our previous work [13], allowing for a coherent comparison between these studies.

A key limitation of our modelling approach is the small sample size (13 patients × 4 muscles), which may reduce the robustness of the findings and limit the generalizability. A common guideline for linear regression models suggests at least 10–15 observations per predictor variable to ensure reliable estimates. Given the inclusion of multiple predictors in our second model, the risk of overfitting increases. Therefore, the interpretation of our regression models should be approached with caution. Future studies with larger cohorts are essential to validate these findings and provide more definitive conclusions.

As aTSC reflects the overall sodium content in a voxel, additional sequences with an inversion‐recovery preparation aim to distinguish signals origins based on relaxation differences between intra‐ and extracellular compartments. However, a clear separation of the intra‐ and extracellular ^23^Na signal is not possible [37]. We refer to ^23^Na images with inversion‐recovery preparation as ICwS maps, as signals from blood vessels are substantially suppressed.

## 6 Conclusions

This study demonstrated the utility of multiparametric MRI in providing new insights into the pathophysiological mechanisms of DMD. While FF increases were not influenced directly by baseline sodium levels, the interaction between ICwS and time revealed its potential role in FF progression. Additionally, persistent elevations of aTSC and ICwS, despite their limited direct correlation with FF changes, reflect underlying ionic disturbances and muscle‐specific adaptations. Further research using ^23^Na and ^1^H MRI is needed to understand the underlying pathophysiological mechanisms and eventually explore their utility in monitoring therapeutic interventions.

## Conflicts of Interest

The authors declare no conflicts of interest.

## Supporting information


**Table S1.** Summary of patient’s status of ambulation and medication for each visit as well as their mutation. Steroid medication is highlighted in bold.
**Table S2.** Fat fraction (FF), water T_2_ (wT_2_), apparent total tissue sodium concentration (aTSC), and intracellular‐weighted sodium signal (ICwS) for controls and patients with DMD at Visit 1. Wilcoxon rank sum tests (WRST) tested equality between control and DMD populations and effect size was calculated using Hedge’s g* (high effect sizes *g* > 0.8 are marked in bold).
**Table S3.** Linear regression model results for sodium concentrations. Analysis included four different muscles, fat fraction (FF), apparent total tissue sodium concentration (aTSC), and intracellular‐weighted sodium signal (ICwS) for controls and patients with DMD at Visit 1.
**Table S4.** Fat fraction (FF), water T_2_ (wT_2_), apparent total tissue sodium concentration (aTSC), and intracellular‐weighted sodium signal (ICwS) for patients with DMD at Visits 2 and 3 and changes between Visits 1 and 2 and between Visits 1 and 3. Wilcoxon signed‐rank tests (WSRT) tested equality muscle‐wise between different visits. The standardized response mean (SRM) assesses the magnitude of change over time (moderate to high values SMR > 0.5 are marked in bold).
**Table S5.** Results from two linear mixed‐effects models assessing changes in fat fraction (FF) over time. The first model evaluates FF changes based on the number of days and muscle group, while the second model examines FF changes with respect to baseline aTSC levels and muscle groups. The third and more complex model investigates the combined effects of disease activity (aTSC, ICwS, and wT_2_) on FF progression.
**Figure S1.** MR‐based pH measurement of the dystrophic muscle. (A) Axial slice of the human calf showing the voxel placement in the gastrocnemius medialis for ^1^H spectroscopy and an exemplary ^1^H spectrum of one DMD patient with the C_2_‐H carnosine at around 8 ppm and H_2_0 at 4.7 ppm. (B) Boxplots representing the pH values of controls (Ctrl, blue) and DMD patients (green) at baseline (v1) and follow‐up visits (v2, v3). pH in DMD patients at baseline was significantly increased compared with the control cohort (Wilcoxon rank‐sum test, *p* < 0.05, marked with *).
**Figure S2.** Apparent tissue sodium concentration (aTSC) and intracellular‐weighted sodium signal (ICwS) values obtained with the inversion‐recovery (IR) sequence, shown both before and after correction for fat fraction (FF) derived from the ^1^H Dixon sequence. Each data point represents an individual muscle from a subject (DMD patients in blue, red and yellow crosses; controls in violet diamonds).
**Figure S3.** Correlation plots of fat fraction with apparent tissue sodium concentration (aTSC) or intracellular‐weighted sodium signal (ICwS) before and after fat fraction correction. Each data point represents an individual muscle from a subject (DMD patients in blue, red and yellow crosses; controls in violet diamonds). Linear correlation lines (in grey) were computed only for patient muscles, with corresponding Spearman correlation coefficients. Following fat fraction correction, the correlation lines show a reduced slope.
**Figure S4.** Sodium distribution (corrected by the fat fraction) along the analysed muscle length of 3.5 cm. (A) Apparent tissue sodium concentration (aTSC) and (B) intracellular‐weighted sodium signal (ICwS) values are plotted across five slices along the proximodistal axis for each of the four analysed muscles. The left slices show the more distal muscle part while the right slices show the proximal muscle part. Each line corresponds to an individual subject: grey for controls and coloured for DMD patients at their first visit. Mean values per slice are shown with a solid black line for DMD and a dashed black line for controls. The average coefficient of variation (CV) across the five slices is also provided for both groups.
**Figure S5.** Quantification of ^23^Na MRI data in dependence of slice selection. Correlation (left) and Bland–Altmann (right) plots of the difference in apparent tissue sodium concentration (aTSC) and intracellular‐weighted sodium signal (ICwS) between the middle slice measurement (slice thickness 7 mm) and the average over the five middle slices (covering 3.5 cm). Each data point represents one individual muscle of an individual subject after fat correction (DMD in blue and control in grey). GM … gastrocnemius medialis, Sol … soleus, TA … tibialis anterior, TP … tibialis posterior; LOA … limits of agreement (equals 1.96 × standard deviation) and % of values.
**Figure S6.** Changes in fat fraction over disease activity at Visit 1. Correlation plots show the difference in fat fraction between Visits 2 and 1 (upper row) and Visits 3 and 1 (lower row) as a function of water T_2_, apparent tissue sodium concentration (aTSC) and intracellular‐weighted sodium signal (ICwS) measured at Visit 1. Each circle represents one individual muscle of a patient with fat fraction < 0.1 at Visit 1, and each diamond represents one individual muscle of a patient with fat fraction > 0.1 at Visit 1. Both muscles with and without changes in fat fraction exhibited water T_2_, aTSC and ICwS increases compared with controls (indicated by horizontal grey line). CTL … control, GM … gastrocnemius medialis, Sol … soleus, TA … tibialis anterior, TP … tibialis posterior.
**Figure S7.** Correlations between disease activity parameters, including Spearman correlation coefficients. The plots illustrate the relationships among water T_2_, apparent tissue sodium concentration (aTSC), intracellular‐weighted sodium signal (ICwS) and pH across all visits. Each point represents an individual muscle from either a patient (coloured) or a control (grey). While water T_2_ and sodium‐related parameters (aTSC and ICwS) exhibit some degree of correlation, no significant correlations were observed for pH with other parameters. CTL … control, GM … gastrocnemius medialis, Sol … soleus, TA … tibialis anterior, TP … tibialis posterior.
