# Supplementary material for: Longitudinal Follow‐Up of Patients With Duchenne Muscular Dystrophy Using Quantitative 23Na and 1H MRI
Source: J Cachexia Sarcopenia Muscle. 2025 Apr 20;16(2):e13812. doi: 10.1002/jcsm.13812 (PMC12009636; doi:10.1002/jcsm.13812)
Supplement: Supplementary file 1 — Table S1. Summary of patient’s status of ambulation and medication for each visit as well as their mutation. Steroid medication is highlighted in bold. Table S2. Fat fraction (FF), water T2 (wT2), apparent total tissue sodium concentration (aTSC), and intracellular‐weighted sodium signal (ICwS) for controls and patients with DMD at Visit 1. Wilcoxon rank sum tests (WRST) tested equality between control and DMD populations and effect size was calculated using Hedge’s g* (high effect sizes g > 0.8 are marked in bold). Table S3. Linear regression model results for sodium concentrations. Analysis included four different muscles, fat fraction (FF), apparent total tissue sodium concentration (aTSC), and intracellular‐weighted sodium signal (ICwS) for controls and patients with DMD at Visit 1. Table S4. Fat fraction (FF), water T2 (wT2), apparent total tissue sodium concentration (aTSC), and intracellular‐weighted sodium signal (ICwS) for patients with DMD at Visits 2 and 3 and changes between Visits 1 and 2 and between Visits 1 and 3. Wilcoxon signed‐rank tests (WSRT) tested equality muscle‐wise between different visits. The standardized response mean (SRM) assesses the magnitude of change over time (moderate to high values SMR > 0.5 are marked in bold). Table S5. Results from two linear mixed‐effects models assessing changes in fat fraction (FF) over time. The first model evaluates FF changes based on the number of days and muscle group, while the second model examines FF changes with respect to baseline aTSC levels and muscle groups. The third and more complex model investigates the combined effects of disease activity (aTSC, ICwS, and wT2) on FF progression. Figure S1. MR‐based pH measurement of the dystrophic muscle. (A) Axial slice of the human calf showing the voxel placement in the gastrocnemius medialis for 1H spectroscopy and an exemplary 1H spectrum of one DMD patient with the C2‐H carnosine at around 8 ppm and H20 at 4.7 ppm. (B) Boxplots representing the pH va [file JCSM-16-e13812-s001.docx]

## Longitudinal follow-up of patients with Duchenne muscular dystrophy

## using quantitative ^23^Na and ^1^H MRI

**Table S1** Summary of patient’s status of ambulation and medication for each visit as well as their mutation. Steroid medication is highlighted in bold.

| **DMD** | **Location of mutation** | **Visit 1** | | | **Visit 2** | | | **Visit 3** | | |
| --- | --- | --- | --- | --- | --- | --- | --- | --- | --- | --- |
|  |  | **Age**  **(years)** | **Ambulant** | **Medication** | **time to v1**  **(weeks)** | **Ambulant** | **Medication** | **time to v1**  **(weeks)** | **Ambulant** | **Medication** |
| 1 | Exon 39 | 6.1 | yes | Ataluren | 27 | yes | **Steroid**, Ataluren | 54 | yes | **Steroid**, Ataluren |
| 2 | Del. Exons 48-50 | 9.8 | yes | **Steroid**, Metformin, Citrulline | 26 | yes | **Steroid**, Metformin, Citrulline | 53 | yes | **Steroid**, Metformin, Citrulline |
| 3 | Exon 51 | 11.4 | no | **Steroid**, Metformin, Citrulline | 27 | no | **Steroid** | 52 | no | **Steroid** |
| 4 | Del. Exons 18-19 | 11.5 | yes | Idebenone | 25 | yes | **Steroid** | 51 | yes | **Steroid** |
| 5 | Del. Exons 48-50 | 11.3 | no | no | 29 | no | no | 62 | no | no |
| 6 | Exon 12 | 5.6 | yes | **Steroid**, Ataluren | NaN | NaN | NaN | NaN | NaN | NaN |
| 7 | Del. Exons 28-43 | 7.2 | yes | no | 23 | yes | **Steroid** | NaN | NaN | NaN |
| 8 | Del. Exons 28-43 | 7.2 | yes | no | 23 | no | no | NaN | NaN | NaN |
| 9 | Exon 41 | 8.6 | yes | **Steroid** | 21 | yes | Ataluren | NaN | NaN | NaN |
| 10 | Del. Exons 45-50 | 7.0 | yes | **Steroid** | 36 | yes | **Steroid**, Eteplirsen | NaN | NaN | NaN |
| 11 | Exon 46 | 5.9 | yes | **Steroid** | 41 | yes | **Steroid** | NaN | NaN | NaN |
| 12 | Del. Exon 45 | 5.0 | yes | no | 26 | yes | **Steroid** | 84 | yes | **Steroid** |
| 13 | Del. Exons 51-52 | 5.4 | yes | no | 33 | yes | no | 85 | yes | **Steroid** |
|  | Mean | 7.8 |  |  | 28.1 |  |  | 62.9 |  |  |
|  | SD | 2.3 |  |  | 5.5 |  |  | 13.9 |  |  |

Del… deletion, NaN… data not available due to loss of follow-up, SD… standard deviation

## MRI data per visit for controls and patients

**Table S2** Fat fraction (FF), water T_2_ (wT_2_), apparent total tissue sodium concentration (aTSC) and intracellular-weighted sodium signal (ICwS) for controls and patients with DMD at visit 1. Wilcoxon rank sum tests (WRST) tested equality between control and DMD populations and effect size was calculated using Hedge’s *g** (high effect sizes *g* > 0.8 are marked in bold).

|  |  |  | Control | | |  | DMD visit 1 | | |  | Hedge's g* |
| --- | --- | --- | --- | --- | --- | --- | --- | --- | --- | --- | --- |
|  | muscle | n | median | Q1 | Q3 | n | median | Q1 | Q3 | p |  |
| FF | soleus | 14 | .026 | .022 | .033 | 13 | .047 | .040 | .095 | <0.001 | **1.12** |
|  | gastrocnemius medialis | 14 | .042 | .037 | .049 | 13 | .104 | .061 | .178 | 0.002 | **1.38** |
|  | tibialis anterior | 14 | .025 | .021 | .030 | 13 | .040 | .028 | .062 | 0.020 | **0.94** |
|  | tibialis posterior | 14 | .033 | .024 | .041 | 13 | .038 | .028 | .057 | 0.215 | 0.57 |
|  | global leg | 14 | .033 | .030 | .034 | 13 | .060 | .040 | .111 | <0.001 | **1.22** |
| wT_2_ | soleus | 14 | 35.5 | 35.2 | 36.2 | 13 | 40.8 | 37.3 | 43.1 | 0.002 | **1.50** |
|  | gastrocnemius medialis | 14 | 34.8 | 34.3 | 36.2 | 13 | 39.2 | 37.6 | 40.3 | <0.001 | **1.69** |
|  | tibialis anterior | 14 | 34.6 | 34.2 | 35.4 | 13 | 39.0 | 36.7 | 41.8 | <0.001 | **1.80** |
|  | tibialis posterior | 14 | 34.8 | 34.1 | 35.1 | 13 | 38.0 | 37.0 | 39.5 | <0.001 | **1.59** |
|  | global leg | 14 | 35.2 | 34.6 | 35.3 | 13 | 39.3 | 37.7 | 41.6 | <0.001 | **1.89** |
| aTSC | soleus | 14 | 15.4 | 14.2 | 15.9 | 11 | 27.5 | 21.9 | 32.8 | <0.001 | **2.41** |
|  | gastrocnemius medialis | 14 | 15.9 | 15.0 | 16.6 | 11 | 25.3 | 20.9 | 28.9 | <0.001 | **2.74** |
|  | tibialis anterior | 14 | 12.9 | 12.0 | 14.0 | 11 | 23.1 | 20.4 | 26.6 | <0.001 | **3.66** |
|  | tibialis posterior | 14 | 13.0 | 11.8 | 14.4 | 11 | 22.1 | 20.2 | 23.9 | <0.001 | **3.90** |
|  | global leg | 14 | 14.0 | 13.6 | 14.9 | 11 | 24.4 | 22.2 | 27.7 | <0.001 | **3.56** |
| ICwS | soleus | 14 | 15.0 | 14.0 | 16.1 | 11 | 23.9 | 23.6 | 27.0 | <0.001 | **3.26** |
|  | gastrocnemius medialis | 14 | 14.6 | 14.2 | 15.4 | 11 | 22.3 | 20.2 | 24.1 | <0.001 | **2.40** |
|  | tibialis anterior | 14 | 12.5 | 12.1 | 12.9 | 11 | 22.9 | 21.5 | 24.6 | <0.001 | **3.90** |
|  | tibialis posterior | 14 | 12.8 | 12.0 | 15.4 | 11 | 22.9 | 21.6 | 24.5 | <0.001 | **3.52** |
|  | global leg | 14 | 13.6 | 13.0 | 15.0 | 11 | 23.2 | 22.1 | 23.7 | <0.001 | **3.93** |
| pH | gastrocnemius medialis | 13 | 7.03 | 7.03 | 7.05 | 11 | 7.07 | 7.04 | 7.11 | 0.028 | **0.94** |

**Table S3** Linear regression model results for sodium concentrations. Analysis included four different muscles, fat fraction (FF), apparent total tissue sodium concentration (aTSC) and intracellular-weighted sodium signal (ICwS) for controls and patients with DMD at visit 1.

|  | aTSC |  |  | ICwS |  |  |
| --- | --- | --- | --- | --- | --- | --- |
| Parameter | Estimate (β) | Standard Error | *p*-value | Estimate (β) | Standard Error | *p*-value |
| Intercept | 13.72 | 0.728 | 1.04E-33 | 13.571 | 0.54 | 1.61E-43 |
| Sick | 10.741 | 0.739 | 8.93E-26 | 9.764 | 0.548 | 6.95E-32 |
| tibialis anterior | Reference | - | - | Reference | - | - |
| tibialis posterior | -0.431 | 0.917 | 0.64 | 0.903 | 0.68 | 0.188 |
| soleus | 3.011 | 0.921 | 0.0015 | 2.497 | 0.683 | 0.00042 |
| gastrocnemius medialis | 2.935 | 0.948 | 0.0026 | 1.691 | 0.703 | 0.0181 |
| FatFraction | -17.299 | 7.079 | 0.0164 | -21.016 | 5.25 | 0.00012 |
| Model Statistics | R^2^ = 0.73, *p* < 0.001 | | | R^2^ = 0.79, *p* < 0.001 | | |

**Table S4** Fat fraction (FF), water T_2_ (wT_2_), apparent total tissue sodium concentration (aTSC) and intracellular-weighted sodium signal (ICwS) for patients with DMD at visit 2 and 3 and changes between visits 1 and 2 and between visits 1 and 3. Wilcoxon signed-rank tests (WSRT) tested equality muscle-wise between different visits. The Standardized Response Mean (SRM) assesses the magnitude of change over time (moderate to high values SMR > 0.5 are marked in bold).

|  |  | DMD visit 2 | | | DMD visits 2-1 | | | | | | DMD visit 3 | | | DMD visits 3-1 | | | | | |  | |  |
| --- | --- | --- | --- | --- | --- | --- | --- | --- | --- | --- | --- | --- | --- | --- | --- | --- | --- | --- | --- | --- | --- | --- |
|  | muscle | median | Q1 | Q3 | median | Q1 | Q3 | *p*-value | SRM | median | | Q1 | Q3 | | median | Q1 | Q3 | *p*-value | SRM | |  |  |
| FF | soleus | .052 | .041 | .146 | .008 | .001 | .023 | .036 | **0.65** | .085 | | .043 | .253 | | .019 | -.001 | .076 | .078 | **0.72** | |  |  |
|  | gastrocnemius medialis | .111 | .045 | .183 | -.001 | -.010 | .024 | .622 | 0.25 | .097 | | .044 | .245 | | .007 | -.011 | .035 | .578 | 0.40 | |  |  |
|  | tibialis anterior | .045 | .031 | .084 | .008 | -.002 | .015 | .110 | **0.51** | .053 | | .030 | .132 | | .002 | -.007 | .034 | .468 | **0.51** | |  |  |
|  | tibialis posterior | .044 | .027 | .069 | .004 | -.003 | .007 | .204 | 0.37 | .053 | | .037 | .075 | | .016 | -.006 | .027 | .219 | **0.58** | |  |  |
|  | *global leg* | *.069* | *.038* | *.125* | *.006* | *.000* | *.013* | *.077* | *0.49* | *.072* | | *.033* | *.118* | | *.013* | *-.006* | *.042* | *.297* | ***0.55*** | |  |  |
| wT2 | soleus | 40.6 | 39.2 | 43.0 | -0.6 | -2.9 | 2.3 | .791 | 0.04 | 38.6 | | 35.7 | 40.1 | | -2.3 | -4.5 | 0.2 | .156 | **0.93** | |  |  |
|  | gastrocnemius medialis | 38.5 | 37.7 | 40.9 | -0.9 | -2.5 | 1.6 | .691 | 0.11 | 37.0 | | 35.7 | 39.0 | | -1.1 | -3.7 | 0.5 | .406 | **0.70** | |  |  |
|  | tibialis anterior | 37.8 | 36.6 | 39.0 | -1.6 | -3.7 | 0.7 | .151 | 0.41 | 37.9 | | 36.0 | 38.3 | | -2.9 | -3.6 | 2.1 | .156 | **0.57** | |  |  |
|  | tibialis posterior | 37.2 | 35.6 | 39.1 | -1.1 | -1.7 | -0.6 | .278 | 0.09 | 37.0 | | 36.8 | 37.0 | | -1.8 | -2.5 | 0.2 | .219 | **0.70** | |  |  |
|  | *global leg* | *38.8* | *37.8* | *39.8* | *-1.2* | *-2.1* | *0.2* | *.266* | *0.14* | *37.4* | | *35.6* | *38.8* | | *-1.8* | *-4.4* | *0.03* | *.219* | ***0.79*** | |  |  |
| aTSC | soleus | 28.1 | 23.3 | 31.0 | -1.0 | -5.0 | 2.8 | .765 | 0.19 | 23.4 | | 18.1 | 27.5 | | -4.2 | -7.4 | -0.9 | .156 | **2.30** | |  |  |
|  | gastrocnemius medialis | 26.9 | 23.3 | 29.8 | 0.9 | -2.9 | 4.6 | .520 | 0.09 | 23.1 | | 18.0 | 23.3 | | -3.8 | -5.6 | -2.2 | .016 | 0.64 | |  |  |
|  | tibialis anterior | 23.2 | 18.2 | 26.2 | -2.7 | -4.8 | 2.8 | .365 | 0.28 | 22.2 | | 18.5 | 23.1 | | -1.7 | -6.0 | 0.0 | .078 | 0.90 | |  |  |
|  | tibialis posterior | 21.9 | 19.5 | 27.2 | 0.3 | -2.7 | 2.6 | .898 | 0.07 | 19.2 | | 17.7 | 21.7 | | -1.9 | -4.8 | 0.9 | .219 | 0.55 | |  |  |
|  | *global leg* | *25.1* | *21.0* | *28.2* | *-0.6* | *-3.8* | *3.7* | *.966* | *0.02* | *22.0* | | *18.0* | *24.2* | | *-2.8* | *-5.7* | *-0.9* | *.047* | ***1.08*** | |  |  |
| ICwS | soleus | 25.0 | 21.3 | 27.8 | -0.6 | -1.9 | 1.7 | .700 | 0.06 | 22.1 | | 19.8 | 25.2 | | -2.1 | -3.2 | 0.7 | .469 | **0.58** | |  |  |
|  | gastrocnemius medialis | 23.4 | 18.6 | 26.2 | 0.9 | -2.5 | 2.4 | .831 | 0.15 | 21.1 | | 13.6 | 23.1 | | -2.9 | -4.6 | 1.1 | .219 | 0.10 | |  |  |
|  | tibialis anterior | 22.4 | 19.2 | 24.6 | -0.9 | -2.8 | -0.2 | .175 | 0.26 | 21.2 | | 16.9 | 23.7 | | -0.8 | -3.2 | 0.9 | .688 | 0.08 | |  |  |
|  | tibialis posterior | 25.0 | 21.1 | 26.5 | 0.5 | -2.4 | 2.5 | .898 | 0.09 | 21.5 | | 19.8 | 23.5 | | -1.4 | -3.6 | 0.7 | .469 | 0.08 | |  |  |
|  | *global leg* | *23.3* | *20.4* | *26.0* | *-0.1* | *-2.1* | *1.3* | *.966* | *0.01* | *21.3* | | *17.9* | *23.1* | | *-2.2* | *-4.2* | *0.1* | *.375* | *0.14* | |  |  |
| pH | gastrocnemius medialis | 7.05 | 7.03 | 7.08 | 0.00 | -0.02 | 0.03 | 1.00 | 0.17 | 7.09 | | 7.07 | 7.10 | | 0.05 | 0.02 | 0.06 | .25 | **1.36** | |  |  |

**Table S5** Results from two linear mixed-effects models assessing changes in fat fraction (FF) over time. The first model evaluates FF changes based on the number of days and muscle group, while the second model examines FF changes with respect to baseline aTSC levels and muscle groups. The third and more complex model investigates the combined effects of disease activity (aTSC, ICwS, and wT_2_) on FF progression.

| Model | Estimate (β) | Standard Error | t-Statistic | *p*-value |
| --- | --- | --- | --- | --- |
| **Model 1: FF ~ Days + Muscle** |  |  |  |  |
| (Intercept) | 0.0638 | 0.0155 | 4.12 | 6.81E-05 |
| Days | 4.55E-05 | 3.97E-05 | 1.15 | 0.254 |
| tibialis posterior | -0.0204 | 0.0197 | -1.04 | 0.302 |
| soleus | 0.038 | 0.0197 | 1.93 | 0.056 |
| gastrocnemius medialis | 0.0618 | 0.0197 | 3.14 | 0.0021 |
| **Model 2: FF ~ 1 + Muscle + Days * aTSC** |  |  |  |  |
| (Intercept) | 0.1411 | 0.0577 | 2.45 | 0.016 |
| Days | -0.00035 | 0.00023 | -1.49 | 0.14 |
| tibialis posterior | -0.0234 | 0.0206 | -1.14 | 0.259 |
| soleus | 0.0412 | 0.0213 | 1.94 | 0.055 |
| gastrocnemius medialis | 0.0623 | 0.0209 | 2.97 | 0.0036 |
| aTSC | -0.00313 | 0.00234 | -1.34 | 0.184 |
| Days * aTSC | 1.67E-05 | 1.00E-05 | 1.67 | 0.098 |
| **Model 3: FF ~ 1+ aTSC * Days + ICwS * Days + wT2 * Days + Muscle** | | | | |
| (Intercept) | 0.57 | 0.14 | 4.09 | 8.48E-05 |
| Days | -0.0010 | 0.0007 | -1.45 | 0.15 |
| tibialis posterior | -0.009 | 0.017 | -0.49 | 0.62 |
| soleus | 0.038 | 0.017 | 2.19 | 0.031 |
| gastrocnemius medialis | 0.029 | 0.018 | 1.63 | 0.11 |
| TSC | 0.0102 | 0.0033 | 3.13 | 0.0023 |
| ICwS | -0.0147 | 0.0032 | -4.59 | 1.25E-05 |
| wT2 | -0.0105 | 0.0046 | -2.27 | 0.025 |
| Days * TSC | -2.33E-06 | 1.52E-05 | -0.15 | 0.88 |
| Days * ICwS | 2.11E-05 | 7.03E-06 | 3.01 | 0.0033 |
| Days * wT2 | 1.55E-05 | 2.41E-05 | 0.64 | 0.52 |

## Data processing: MR-based pH evaluation

**
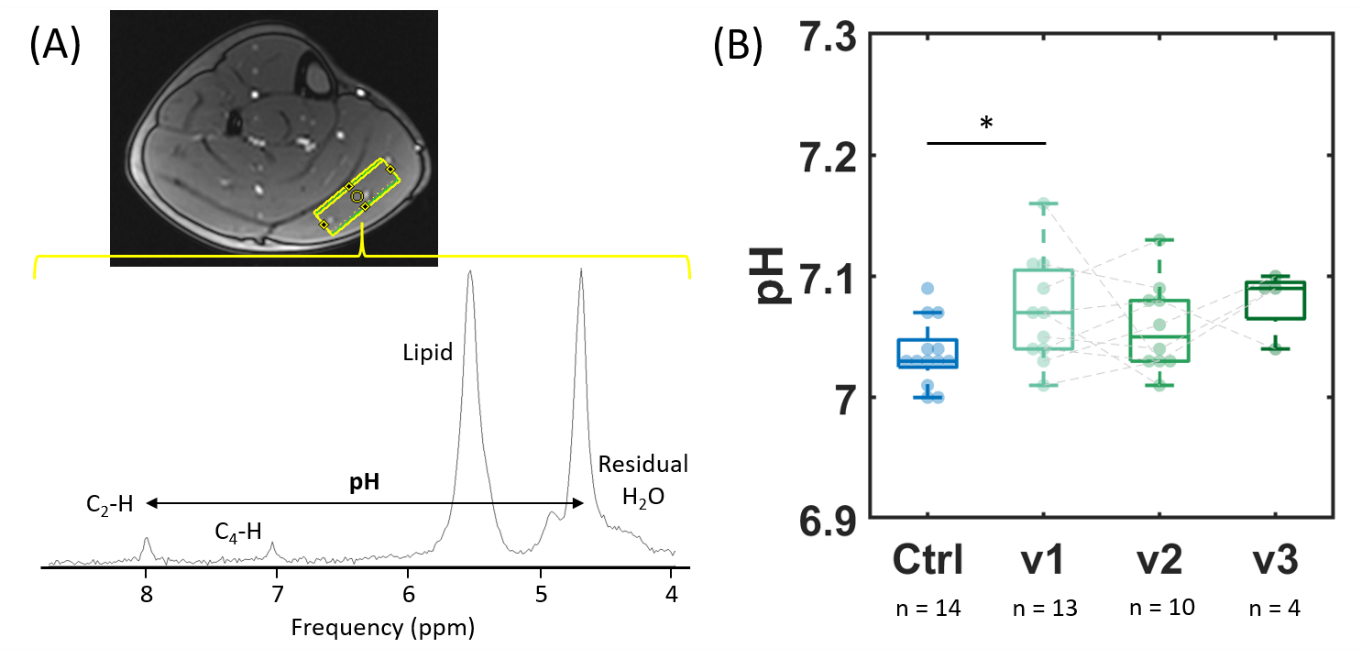
**

**Figure S1** MR-based pH measurement of the dystrophic muscle. (A) Axial slice of the human calf showing the voxel placement in the gastrocnemius medialis for ^1^H spectroscopy and an exemplary ^1^H spectrum of one DMD patient with the C_2_-H carnosine at around 8 ppm and H_2_0 at 4.7 ppm. (B) Boxplots representing the pH values of controls (Ctrl, blue) and DMD patients (green) at baseline (v1) and follow-up visits (v2, v3). pH in DMD patients at baseline was significantly increased compared to the control cohort (Wilcoxon rank-sum test, *p* < 0.05, marked with *).

## Data processing: Impact of fat fraction correction on ^23^Na MRI quantification


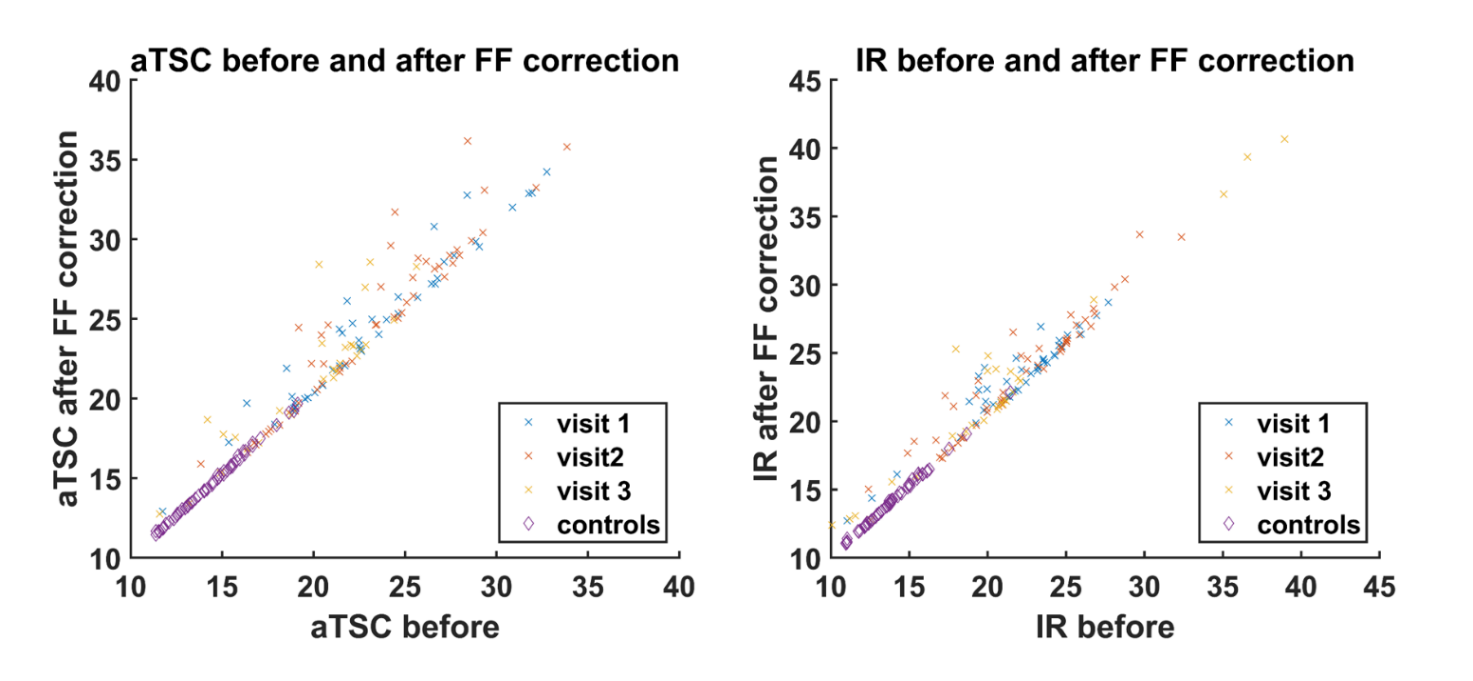


**Figure S2** Apparent tissue sodium concentration (aTSC) and intracellular-weighted sodium signal (ICwS) values obtained with the inversion-recovery (IR) sequence, shown both before and after correction for fat fraction (FF) derived from the ^1^H Dixon sequence**.** Each data point represents an individual muscle from a subject (DMD patients in blue, red, and yellow crosses; controls in violet diamonds).

**
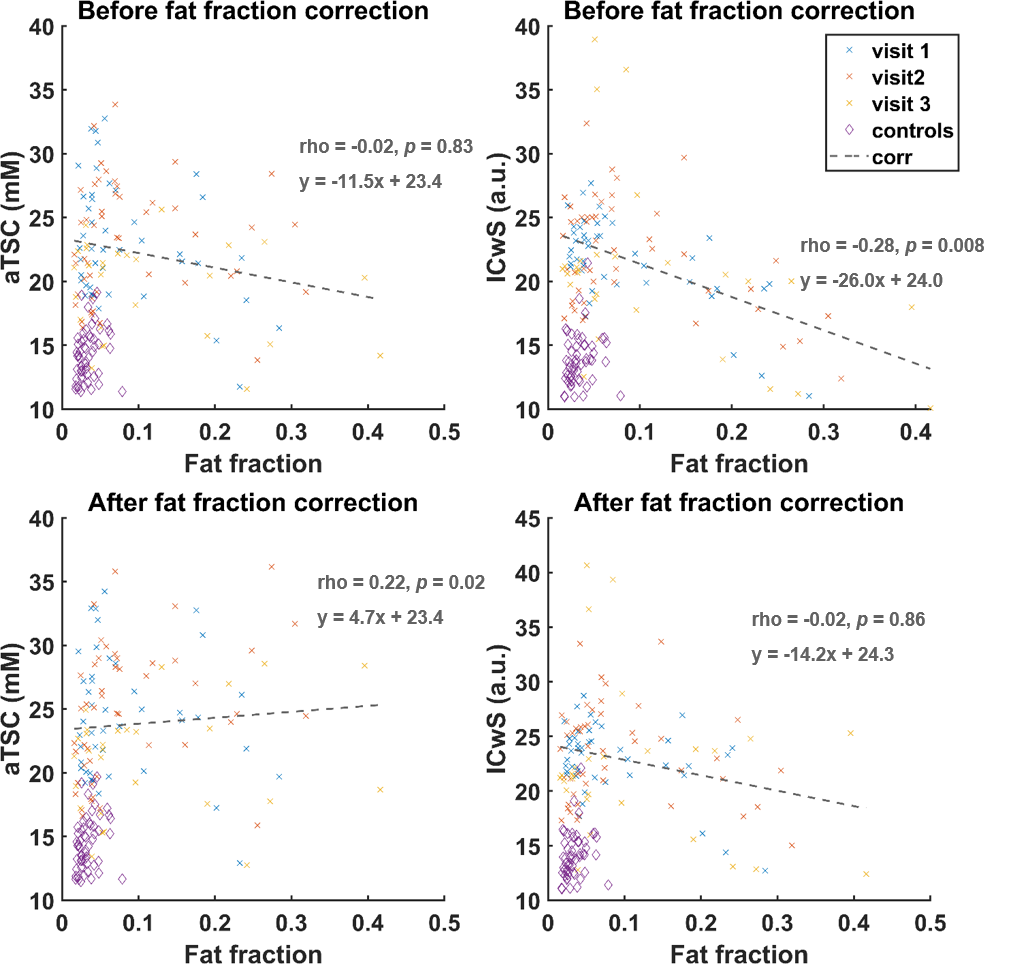
**

**Figure S3** Correlation plots of fat fraction with apparent tissue sodium concentration (aTSC) or intracellular-weighted sodium signal (ICwS) before and after fat fraction correction. Each data point represents an individual muscle from a subject (DMD patients in blue, red, and yellow crosses; controls in violet diamonds). Linear correlation lines (in grey) were computed only for patient muscles, with corresponding Spearman correlation coefficients. Following fat fraction correction, the correlation lines show a reduced slope.

## Data processing: ^23^Na MRI quantification as function of slice selection


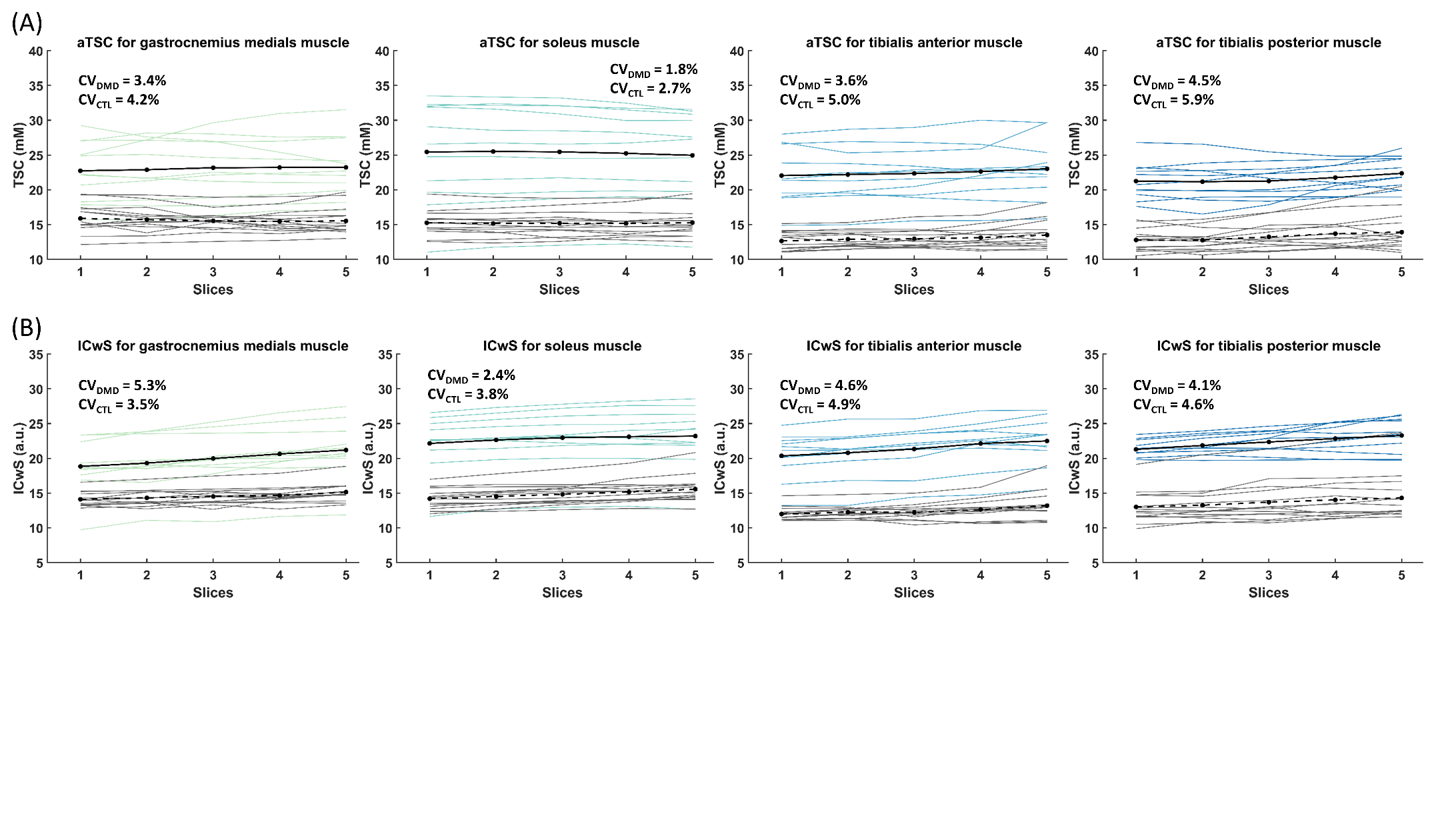


**Figure S4** Sodium distribution (corrected by the fat fraction) along the analyzed muscle length of 3.5 cm. (A) Apparent tissue sodium concentration (aTSC) and (B) intracellular-weighted sodium signal (ICwS) values are plotted across five slices along the proximodistal axis for each of the four analyzed muscles. The left slices show the more distal muscle part while the right slices show the proximal muscle part. Each line corresponds to an individual subject: gray for controls and colored for DMD patients at their first visit. Mean values per slice are shown with a solid black line for DMD and a dashed black line for controls. The average coefficient of variation (CV) across the five slices is also provided for both groups.


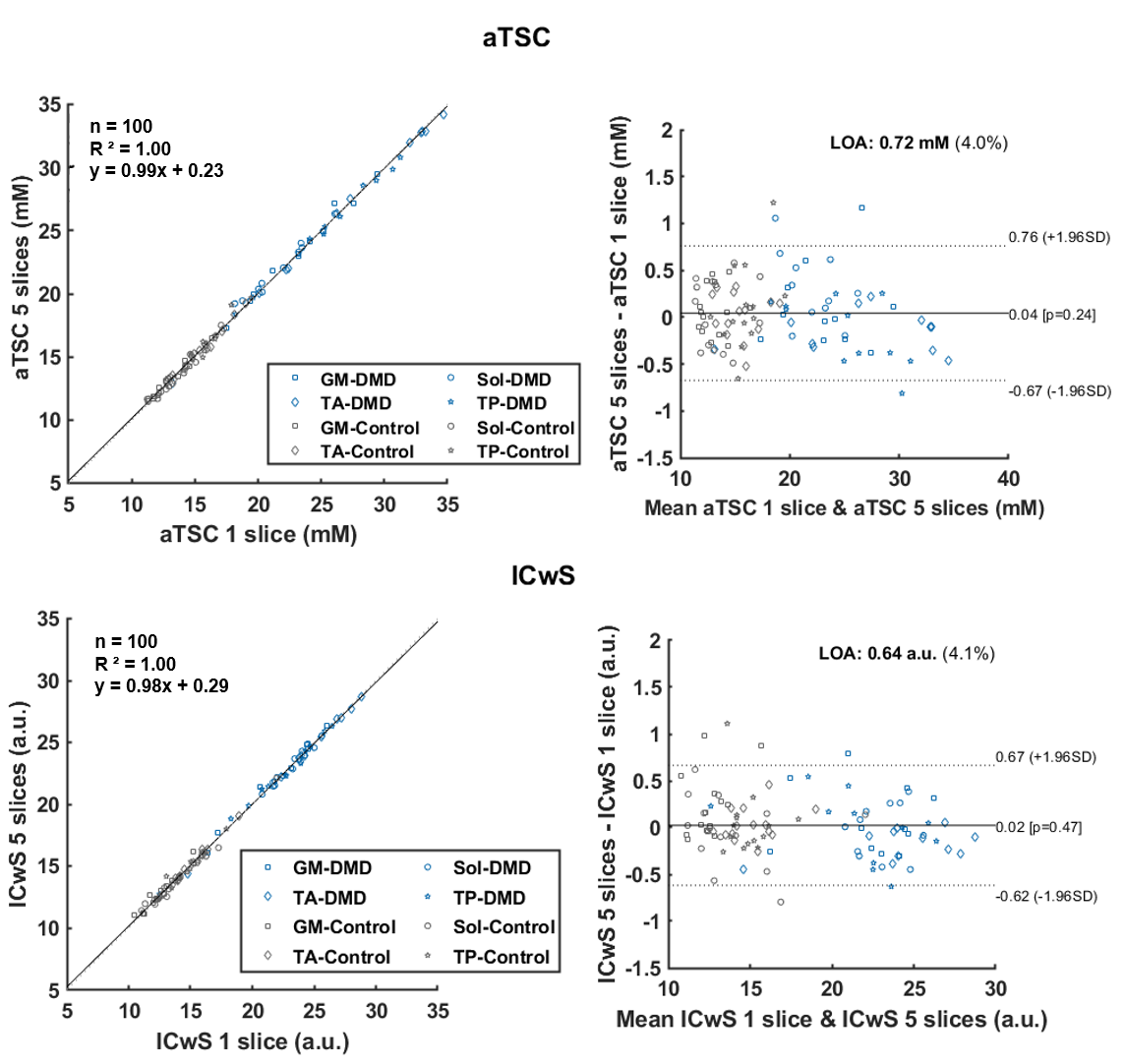


**Figure S5** Quantification of ^23^Na MRI data in dependence of slice selection. Correlation (left) and Bland-Altmann (right) plots of the difference in apparent tissue sodium concentration (aTSC) and intracellular-weighted sodium signal (ICwS) between the middle slice measurement (slice thickness 7 mm) and the average over the five middle slices (covering 3.5 cm). Each data point represents one individual muscle of an individual subject after fat correction (DMD in blue and control in grey). GM… gastrocnemius medialis, Sol… soleus, TA… tibialis anterior, TP… tibialis posterior; LOA… limits of agreement (equals 1.96×standard deviation) and % of values

**
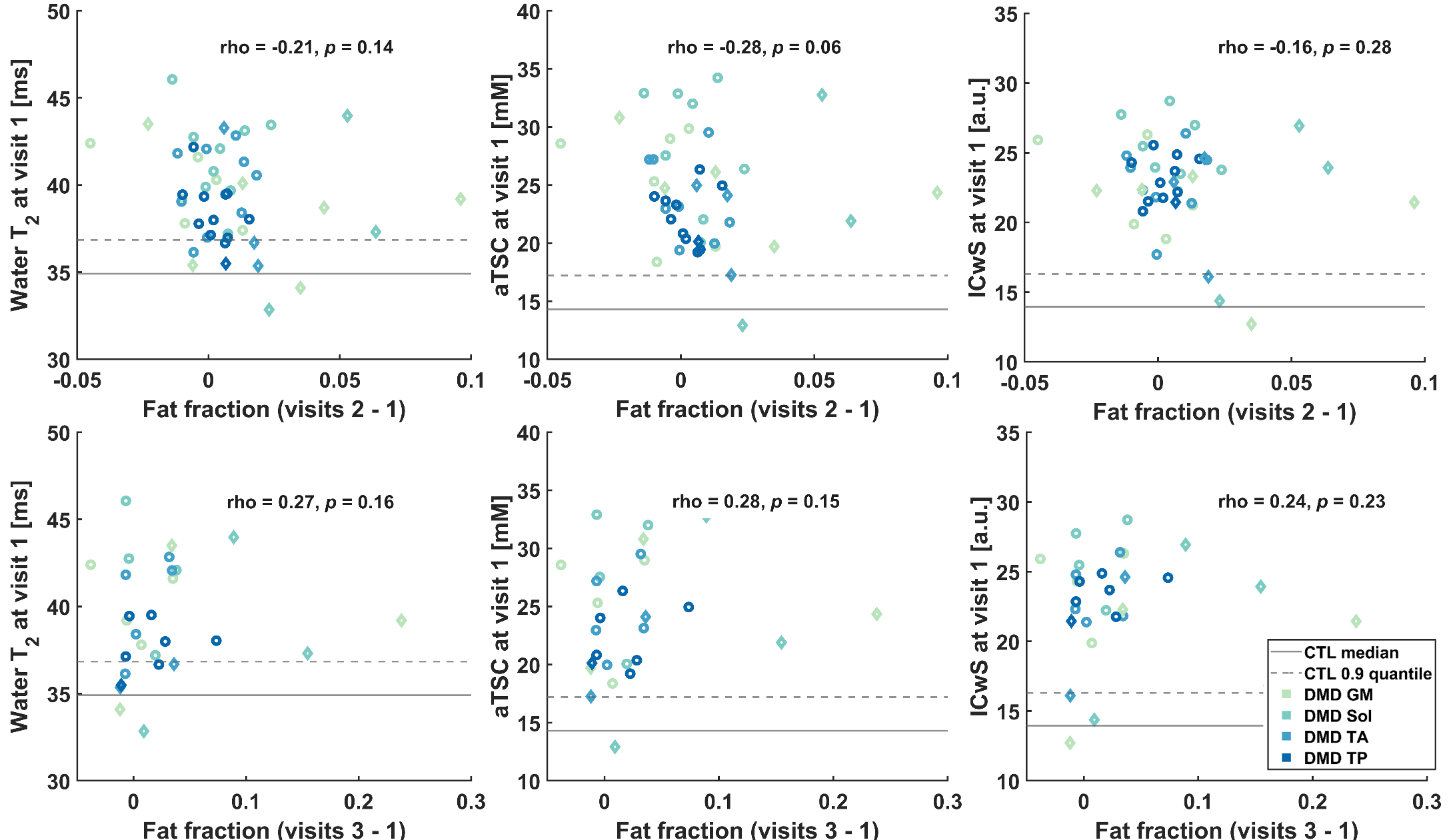
Figure S6** Changes in fat fraction over disease activity at visit 1. Correlation plots show the difference in fat fraction between visits 2 and 1 (upper row) and visits 3 and 1 (lower row) as a function of water T_2_, apparent tissue sodium concentration (aTSC), and intracellular-weighted sodium signal (ICwS) measured at visit 1. Each circle represents one individual muscle of a patient with fat fraction < 0.1 at visit 1, and each diamond represents one individual muscle of a patient with fat fraction > 0.1 at visit 1. Both muscles with and without changes in fat fraction exhibited water T_2_, aTSC, and ICwS increases compared to controls (indicated by horizontal grey line). CTL… control, GM… gastrocnemius medialis, Sol… soleus, TA… tibialis anterior, TP… tibialis posterior

**
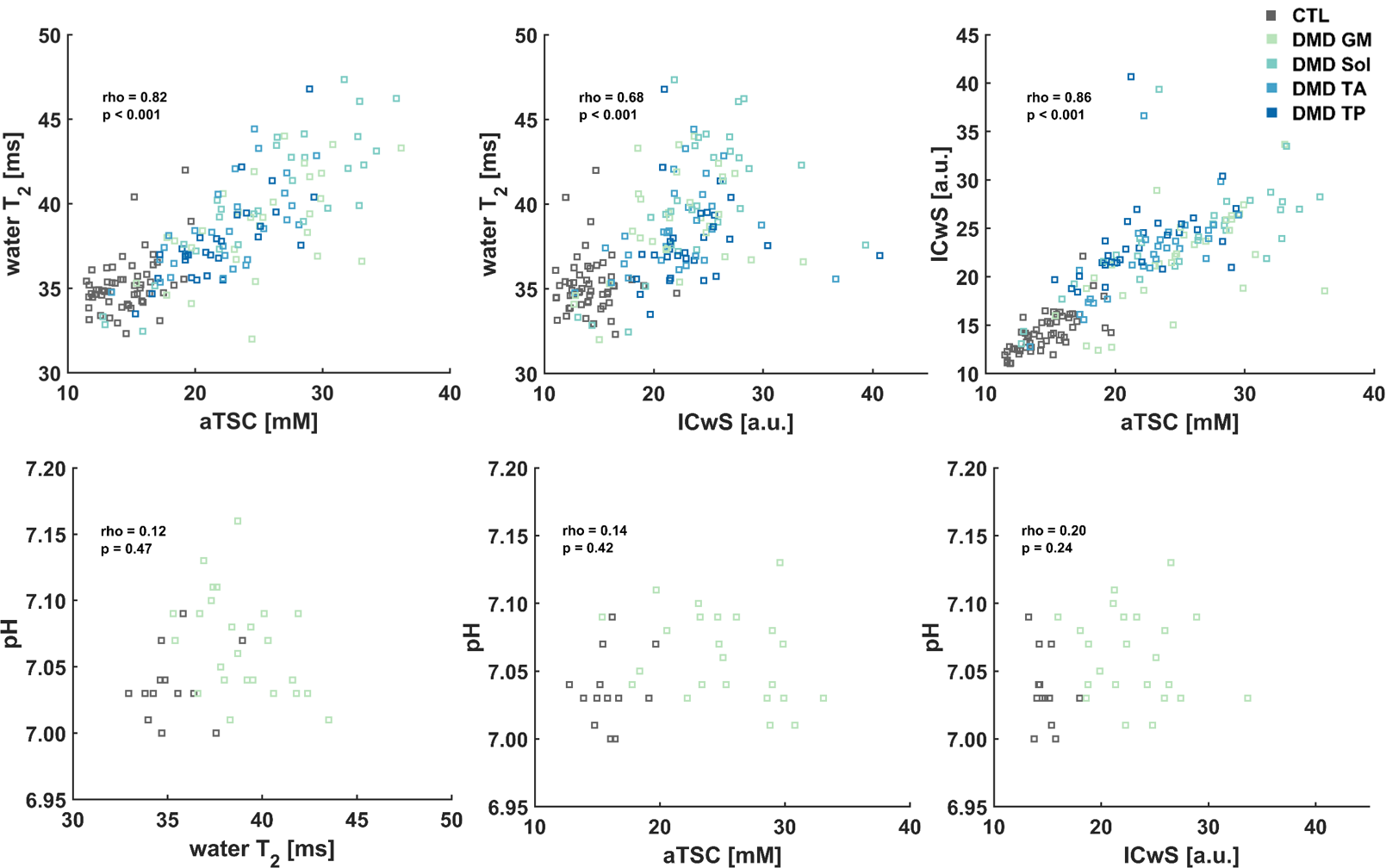
**

**Figure S7** Correlations between disease activity parameters, including Spearman correlation coefficients. The plots illustrate the relationships among water T_2_, apparent tissue sodium concentration (aTSC), intracellular-weighted sodium signal (ICwS), and pH across all visits. Each point represents an individual muscle from either a patient (colored) or a control (gray). While water T_2_ and sodium-related parameters (aTSC and ICwS) exhibit some degree of correlation, no significant correlations were observed for pH with other parameters. CTL… control, GM… gastrocnemius medialis, Sol… soleus, TA… tibialis anterior, TP… tibialis posterior

## Histopathological evidence for sodium hypothesis

Elevated sodium levels in biopsied muscles of DMD patients were first reported in 1952 by Horvath et al., and later confirmed in *mdx* mice and patients through microelectrode and Na^+^-dye measurements (Dunn et al. 1993 and 1995, Hirn et al. 2008, Miles et al. 2011, Altamirano et al. 2014, Burr et al. 2014).

- Horvath, B., L. Berg, D.J. Cummings, and G.M. Shy. 1955. Muscular dystrophy: cation concentrations in residual muscle. J. Appl. Physiol. 8:22–30. <https://doi.org/10.1152/jappl.1955.8.1.22>
- Dunn, J.F., N. Bannister, G.J. Kemp, and S.J. Publicover. 1993. Sodium is elevated in mdx muscles: ionic interactions in dystrophic cells. J. Neurol. Sci. 114:76–80. <https://doi.org/10.1016/0022-510X(93)90052-Z>
- Dunn, J.F., K.A. Burton, and M.J. Dauncey. 1995. Ouabain sensitive Na+/K+-ATPase content is elevated in mdx mice: implications for the regulation of ions in dystrophic muscle. J. Neurol. Sci. 133:11–15. <https://doi.org/10.1016/0022-510X(95)00167-Z>
- Hirn, C., G. Shapovalov, O. Petermann, E. Roulet, and U.T. Ruegg. 2008. Nav1.4 deregulation in dystrophic skeletal muscle leads to Na+ overload and enhanced cell death. J. Gen. Physiol. 132:199–208. <https://doi.org/10.1085/jgp.200810024>
- Miles, M.T., E. Cottey, A. Cottey, C. Stefanski, and C.G. Carlson. 2011. Reduced resting potentials in dystrophic (mdx) muscle fibers are secondary to NF-κB-dependent negative modulation of ouabain sensitive Na+-K+ pump activity. J. Neurol. Sci. 303:53–60. <https://doi.org/10.1016/j.jns.2011.01.015>
- Altamirano, F., C.F. Perez, M. Liu, J. Widrick, E.R. Barton, P.D. Allen, J.A. Adams, and J.R. Lopez. 2014. Whole body periodic acceleration is an effective therapy to ameliorate muscular dystrophy in mdx mice. PLoS One. 9:e106590. <https://doi.org/10.1371/journal.pone.0106590>
- Burr, A.R., and J.D. Molkentin. 2015. Genetic evidence in the mouse solidifies the calcium hypothesis of myofiber death in muscular dystrophy. Cell Death Differ. 22:1402–1412. <https://doi.org/10.1038/cdd.2015.65>
